# Zika exposure during pregnancy and the risk of hospitalisation and death due to community-acquired pneumonia in early childhood: a nationwide registry-based cohort study in Brazil

**DOI:** 10.1016/j.lana.2026.101649

**Published:** 2026-09-30

**Authors:** Everton Falcão de Oliveira, Thiago Cerqueira-Silva, João Guilherme G. Tedde, Bruno Holsback Uesato, José Victor Bortolotto Bampi, Elizabeth B. Brickley, Maria Gloria Teixeira, Mauricio L. Barreto, Enny S. Paixão

**Affiliations:** a Faculdade de Medicina, Universidade Federal de Mato Grosso do Sul, Campo Grande, Brazil; b Programa de Pós-Graduação em Doenças Infecciosas e Parasitárias, Universidade Federal de Mato Grosso do Sul, Campo Grande, Brazil; c Faculty of Epidemiology and Population Health, London School of Hygiene & Tropical Medicine, London, United Kingdom; d Laboratório de Medicina e Saúde Pública de Precisão, Fundação Oswaldo Cruz, Salvador, Brazil; e Centro de Integração de Dados e Conhecimentos para Saúde (Cidacs), Fundação Oswaldo Cruz, Salvador, Brazil; f Departamento de Oftalmologia, Faculdade de Medicina, Universidade de São Paulo, São Paulo, Brazil; g Coordenadoria de Estatísticas Vitais, Secretaria Municipal de Saúde de Campo Grande, Campo Grande, Brazil; h Colorado School of Public Health, University of Colorado Anschutz, Aurora, Colorado, United States of America; i Instituto de Saúde Coletiva, Universidade Federal da Bahia, Salvador, Brazil

**Keywords:** Zika virus, Prenatal exposure delayed effects, Congenital zika syndrome, Community-acquired pneumonia, Hospitalisation, Mortality

## Abstract

**Background:**

Childhood pneumonia remains a leading cause of morbidity and mortality worldwide. Prenatal Zika virus (ZIKV) infection has been associated with severe outcomes such as Congenital Zika Syndrome (CZS), linked to excess hospitalisations. However, the long-term respiratory consequences of prenatal ZIKV exposure across the full clinical spectrum, including children without CZS, remain poorly understood. We aimed to examine the association between prenatal ZIKV exposure and community-acquired pneumonia-related hospitalisation or death during childhood.

**Methods:**

We conducted a population-based cohort study of live-born singletons in Brazil (January 2015–November 2018). We applied three complementary approaches under a triangulation framework, to strengthen confidence in the observed associations: IPTW-weighted Cox regression, a within-sibling stratified Cox model, and a negative control outcome analysis. Separate analyses compared ZIKV-exposed children with and without CZS to unexposed children.

**Findings:**

We included 4,108,409 unexposed children, 3900 ZIKV-exposed without CZS, and 1786 with CZS. Female sex accounted for 48.8%, 46.7%, and 53.1% across the three groups; the majority in each were of Brown/*Pardo* maternal race/ethnicity (64.6%, 67.8%, and 72.4%). By 42 months, cumulative incidence of pneumonia-related hospitalisation or death was 15.6% (95% CI 13.7–17.5) among children with CZS, versus 3.9% (95% CI 3.2–4.7) among ZIKV-exposed children without CZS and 4.6% (95% CI 4.6–4.7) among unexposed children. In IPTW-weighted models, CZS was associated with a fourfold increased risk (aHR 4.12, 95% CI 3.64–4.66), consistent with the sibling comparison (aHR 4.15, 95% CI 1.62–10.64). Prenatal ZIKV exposure without CZS was not associated with the outcome across any approach. Negative control analyses suggested limited residual confounding.

**Interpretation:**

Children exposed to ZIKV in utero without CZS had a risk of pneumonia-related morbidity and mortality comparable to unexposed children, a reassuring finding for most exposed children. An increased risk was associated with prenatal ZIKV exposure only among children with CZS. These findings underscore the need for long-term follow-up and targeted respiratory prevention strategies for children with CZS.

**Funding:**

10.13039/100010269Wellcome Trust, 10.13039/501100000288Royal Society, and 10.13039/501100003593CNPq.

## Resumo

**Contexto:**

A pneumonia adquirida na comunidade permanece como uma das principais causas de morbidade e mortalidade em crianças no mundo. A infecção pré-natal pelo vírus Zika (ZIKV) tem sido associada a desfechos graves, como a Síndrome Congênita do Zika (SCZ), que está associado ao aumento hospitalizações entre crianças com esta síndrome. Contudo, as consequências respiratórias tardias decorrentes da exposição pré-natal ao ZIKV, incluindo em crianças nascidas sem SCZ, permanecem pouco compreendidas.

**Métodos:**

Foi realizado um estudo de coorte de base populacional com nascidos vivos de gestações únicas no Brasil (janeiro de 2015 a novembro de 2018) para estimar o efeito total da exposição pré-natal ao ZIKV sobre a hospitalização e/ou óbito por pneumonia adquirida na comunidade durante a infância. Três abordagens complementares foram utilizadas na perspectiva da triangulação de evidências para fortalecer a inferência causal: regressão de Cox ponderada por IPTW, modelo de Cox estratificado com comparação entre irmãos e análise de desfecho de controle negativo. Análises separadas compararam crianças expostas ao ZIKV, com e sem SCZ, a crianças não expostas.

**Resultados:**

Um total de 4.108.409 crianças não expostas, 3.900 crianças expostas ao ZIKV sem SCZ e 1.786 com SCZ foram analisadas. Aos 42 meses, a incidência acumulada de hospitalização e/ou óbito por pneumonia foi de 15·6% (IC 95% 13·7–17·5) entre as crianças com SCZ, enquanto entre as crianças expostas ao ZIKV sem SCZ e as não expostas, os riscos acumulados foram 3·9% (IC 95% 3·2–4·7) e 4·6% (IC 95% 4·6–4·7), respectivamente. Nos modelos ponderados por IPTW, a SCZ associou-se a um risco quatro vezes maior (aHR 4·12, IC 95% 3·64–4·66), consistente com a comparação entre irmãos (aHR 4·15, IC 95% 1·62–10·64). A exposição pré-natal ao ZIKV sem SCZ não se associou a aumento de risco em nenhuma das abordagens analíticas. As análises de controle negativo sugeriram confundimento residual limitado.

**Interpretação:**

Neste estudo, a exposição pré-natal ao ZIKV aumentou a morbidade e a mortalidade por pneumonia apenas entre as crianças com SCZ; as crianças expostas sem SCZ apresentaram riscos comparáveis aos das não expostas. Esses achados reforçam a necessidade de seguimento de longo prazo e de estratégias direcionadas de prevenção respiratória para crianças com SCZ.

**Financiamento:**

Wellcome Trust, Royal Society, CNPq.

**Research in context**

**Evidence before this study**

Childhood pneumonia is a leading cause of morbidity and mortality worldwide, and children with complex medical conditions arising from prenatal infections are at elevated risk. To identify existing evidence on respiratory outcomes and broader morbidity and mortality among children with prenatal Zika virus (ZIKV) exposure, we searched PubMed using two complementary strategies. The first combined terms for Zika virus and prenatal exposure with respiratory outcomes and pneumonia ((“Zika Virus” [MeSH] OR “Zika Virus Infection” [MeSH] OR “Zika” OR “ZIKV”) AND (“Prenatal Exposure Delayed Effects” [MeSH] OR “prenatal exposure” OR “congenital” OR “in utero”) AND (“Congenital Zika Syndrome” OR “CZS” OR “Microcephaly” [MeSH]) AND (“Pneumonia” [MeSH] OR “Respiratory Tract Infections” [MeSH] OR “community acquired pneumonia” OR “lower respiratory tract infection” OR “Hospitalisation” [MeSH])). The second replaced respiratory terms with broader morbidity and mortality outcomes ((“Hospitalisation”[MeSH] OR “Hospital Mortality”[MeSH] OR “Child Mortality”[MeSH] OR “mortality” OR “morbidity” OR “cohort study”[MeSH])). Both searches covered the period from January 2015 to May 2026 and were conducted on 20 May 2026, with no language restrictions, yielding 14 and 71 results respectively; after deduplication, seven articles were considered directly relevant. Existing evidence establishes that children with Congenital Zika Syndrome (CZS) face a higher risk of early death. A nationwide Brazilian cohort study found that CZS was associated with an elevenfold increased all-cause mortality risk compared to unexposed children through 36 months of age, with deaths attributable to neurological, respiratory, and infectious causes. A subsequent study extending follow-up to five years confirmed a more than 13-fold increased mortality risk, with pneumonia and urinary tract infections identified as common causes of hospitalisation. A record-linkage study further characterised causes of death, finding that respiratory and infectious diseases were over represented. A nationwide cohort study also found that children with CZS had elevated rates of all-cause hospitalisation throughout early childhood, with respiratory and infectious causes accounting for much of that excess. Two recent narrative reviews note that pneumonia is among the most common causes of hospitalisation in children with CZS, and point to immune dysregulation and impaired respiratory control, consequences of ZIKV neurotropism and its interference with foetal organogenesis, as plausible biological mechanisms. Evidence on children prenatally exposed to ZIKV without CZS has focused on neurodevelopmental outcomes; no study has examined whether this larger group faces an elevated risk of community-acquired pneumonia compared to unexposed children.

**Added value of this study**

Our study provides the first population-based evidence on community-acquired pneumonia-related hospitalisation and death specifically as an outcome of prenatal ZIKV exposure, examining the full clinical spectrum, including those with and without CZS, against a comparison group of over four million unexposed children across Brazil. By applying three complementary analytical approaches (IPTW-weighted Cox regression, within-sibling stratified models, and a negative control outcome analysis), we were able to strengthen confidence in the observed associations beyond what observational designs alone typically allow. Our findings show that CZS was associated with a fourfold increased risk of pneumonia-related morbidity and mortality, consistent with the excess all-cause hospitalisation and mortality reported by prior studies, and now attributable specifically to community-acquired pneumonia. We show that prenatal ZIKV exposure without CZS was not associated with any increase in pneumonia-related morbidity and mortality, with risks in this group comparable to those of unexposed children across all analytical approaches.

**Implications of all the available evidence**

This study adds to the existing evidence on the long-term consequences of prenatal ZIKV exposure by providing the first population-based estimates of community-acquired pneumonia-related morbidity and mortality across the full clinical spectrum of affected children. Our findings indicate that the excess respiratory burden associated with prenatal ZIKV exposure is concentrated among children with CZS, while those without congenital manifestations face risks comparable to unexposed children. These results reinforce the need for long-term clinical follow-up and targeted respiratory prevention strategies for children with CZS. Whether specific measures such as immunisation against respiratory pathogens can mitigate this risk warrants dedicated evaluation. Future research should investigate the biological pathways, including immune dysregulation and impaired respiratory neural control, that drive the elevated pneumonia risk in this population, as well as the potential benefits of early intervention programmes for children with CZS in endemic settings.

## Introduction

Childhood pneumonia remains the leading infectious cause of morbidity and mortality among children under five years of age worldwide, accounting for more than 150 million cases annually, of which a substantial proportion progress to severe disease requiring hospitalisation^1^^,^^2^ and approximately 0.9 to 1 million deaths annually in recent years.^1^^,^^3^^,^^4^ The burden of pneumonia-related morbidity and mortality is disproportionately concentrated in early childhood, particularly during the first two years of life.^5^^,^^6^ This heightened vulnerability reflects a combination of biological vulnerability (e.g., limited acquired immunity, small airways) and early-life determinants, including perinatal exposures (e.g., maternal smoking during pregnancy) and post-natal exposures (e.g., lack of exclusive breastfeeding, household crowding, and second-hand smoke exposure). Underlying child health conditions, such as congenital anomalies also constitute an important component of this biological susceptibility and are also independently associated with an increased risk of severe disease, intensive care admission, and prolonged hospitalisation.^7^^,^^8^

Prenatal Zika virus (ZIKV) infection emerged as a major public health concern due to its adverse outcomes, most notably Congenital Zika Syndrome (CZS), characterised by microcephaly and other central nervous system abnormalities.^9^^,^^10^ Children with CZS experience substantial morbidity and mortality beyond the neonatal period, including high rates of hospitalisations due to respiratory and other infectious diseases,^11^ which contribute to excess early childhood mortality.^12^, ^13^, ^14^ The neurological impairment that characterises the syndrome may predispose to respiratory infection, through dysphagia, recurrent aspiration, and impaired airway protection.^9^^,^^10^ In addition, children with CZS have been shown to exhibit marked and persistent systemic inflammatory imbalance, which may further increase susceptibility to severe infections, including pneumonia.^15^^,^^16^ Similarly, accumulating evidence indicates that children exposed to ZIKV in utero who do not develop CZS may also develop long-term adverse outcomes, including neurodevelopmental delays in language, cognition and motor domains.^17^, ^18^, ^19^

Although existing prospective cohorts have included children across the full spectrum of prenatal ZIKV exposure,^20^ these studies have been largely based on relatively small samples recruited in selected clinical or research settings, and with limited follow-up duration, restricting their capacity to capture population-level estimates of long-term health outcomes across Brazil. Most of the studies have emphasised the need for long-term surveillance of neurological outcomes in children exposed to ZIKV,^18^ with scarce evidence about the respiratory health. This gap is particularly relevant given consistent evidence that infectious and respiratory diseases constitute a major component of the morbidity and mortality burden among children with CZS.^11^, ^12^, ^13^, ^14^ Moreover, evidence remains scarce regarding children exposed in utero who do not present with congenital manifestations at birth. In this study, we investigate the association between prenatal ZIKV exposure and community-acquired pneumonia-related morbidity and mortality in childhood using a large population-based birth cohort derived from national surveillance and administrative data in Brazil. We focus on community-acquired pneumonia and exclude aspiration pneumonitis, which, although clinically relevant in CZS given the associated dysphagia, reflects a distinct pathophysiological mechanism and could otherwise lead to outcome misclassification in this study.

## Methods

### Study design and period

We conducted a population-based cohort study using three complementary analytic approaches, each relying on different assumptions and sources of potential bias^21^ to compare children born to mothers with and without notified ZIKV infection during pregnancy. This cohort study adhered to the Strengthening the Reporting of Observational Studies in Epidemiology (STROBE) reporting guidelines (Supplementary Material in Supplementary Appendix 1).

The study period spanned from January 1, 2015, to November 30, 2018, encompassing the peak and subsequent decline of the ZIKV epidemic in Brazil.^22^ Follow-up began at birth and continued until the first occurrence of the respective outcome, with censoring at death from other causes or at the end of follow-up (November 30, 2018). Children were followed from birth up to approximately four years of age, depending on their birth date. Data analyses were conducted between September 2025 and December 2025.

### Data sources

We used data from the CIDACS Birth Cohort,^23^ a large-scale, population-based Brazilian cohort created through the linkage of administrative health and social registries. The cohort comprises live-born children from mothers registered in the Unified Registry for Social Programs (*Cadastro Único para Programas Sociais*, CadUnico), Brazil's national registry for families applying for social assistance,^24^ and is defined through record linkage between CadUnico and the Live Birth Information System (*Sistema de Informação sobre Nascidos Vivos*, SINASC). During the study period (January 2015–November 2018), SINASC recorded 11,507,017 live births in Brazil, of which 5,695,179 (49.5%) were born to mothers registered in CadUnico and therefore constituted the CIDACS Birth Cohort used in this study. SINASC covers nearly all live births in Brazil and provides detailed information on maternal, newborn, and pregnancy-related characteristics based on data recorded in the live birth certificate, which is completed by the health professional who attended or assisted the delivery.^25^^,^^26^ CadUnico provides individual- and household-level socioeconomic information collected at the time of registration for social programs.

Information on exposure and outcomes was obtained through further data linkage. Data on reported infections during pregnancy were obtained from the Notifiable Diseases Information System (*Sistema de Informação de Agravos de Notificação*, SINAN), a national registry that compiles mandatory notifications of infectious and non-infectious conditions. Reported cases undergo epidemiological investigation and are classified based on laboratory confirmation or clinical–epidemiological criteria, particularly during epidemic periods.^27^ From SINAN, we used notification records of maternal Zika virus infection to define exposure, and records of dengue and chikungunya to exclude children exposed to these other arboviruses during pregnancy.

We also used data from the Public Health Events Registry (*Registro de Eventos em Saúde Pública*, RESP-Microcefalia).^28^ Data from RESP-Microcefalia were used to identify CZS cases based on final case classification (confirmed, probable, or ruled out), defined according to the Brazilian Ministry of Health surveillance protocol in force during the study period,^28^ as well as information on other antenatal exposures, including syphilis and toxoplasmosis infections.

Mortality data were retrieved from the Mortality Information System (*Sistema de Informação sobre Mortalidade*, SIM), which systematically records deaths in Brazil and is based on standardised death certificates completed by the attending physician or legally responsible health professional.^29^ This system includes information on sociodemographic characteristics, place of occurrence and residence, and cause of death coded according to the 10th revision of the International Classification of Diseases (ICD-10). From SIM, we extracted data on the underlying cause of death and date of death.

Information on hospitalisation due to community-acquired pneumonia was obtained from the Hospital Information System of the Brazilian Unified Health System (*Sistema de Informações Hospitalares do Sistema Único de Saúde*, SIH/SUS), which records hospital admissions financed by the SUS and is estimated to cover approximately 70% of all hospitalisations in Brazil. This system includes data on admission dates, procedures performed, length of stay, and diagnoses coded according to the ICD-10.^30^ From SIH/SUS, we obtained admission dates and primary causes of hospitalisation.

### Linkage

Data linkage procedures were conducted using CIDACS-Record Linkage,^31^ a validated record linkage tool developed to integrate large-scale Brazilian administrative databases. This tool combines indexing and searching algorithms to efficiently identify potential matches across datasets and performs pairwise comparisons of candidate records, ranking them according to linkage scores and retaining the highest-scoring pair as the most likely match.^31^ Records from the CIDACS Birth Cohort were linked to data from SINAN, RESP-Microcefalia, SIM, and SIH/SUS. Linkage was performed using multiple personal identifiers, including maternal name, maternal date of birth or age, place of residence, and child's date of birth, depending on data availability in each source. The accuracy of the linkage process has been previously evaluated through manual review of randomly selected record pairs stratified by linkage score, with sensitivity and specificity consistently exceeding 90% across linked datasets, including linkages between SINASC and SIM, SINASC and SIH/SUS, and SINASC and SINAN.^31^ All linkage procedures were carried out at CIDACS within a secure data environment,^23^^,^^32^ in compliance with Brazilian ethical, legal, and data protection regulations.^33^

### Participants

We included all singleton live-born children in the CIDACS Birth Cohort whose mothers were aged 15–49 years at the time of delivery. We excluded records with missing information on gestational age or the child's sex, as well as those with implausible values for birth weight (<500 g or >6000 g) or gestational age outside the range of viability and clinically plausible limits (<22 or >42 weeks). Records with inconsistent event dates (such as death recorded before birth or before hospital admission) were also excluded. To minimise potential confounding by other arthropod-borne viral infections and other congenital infections, we excluded children whose mothers had a confirmed chikungunya virus (CHIKV) or dengue virus (DENV) infection during pregnancy, as recorded in the SINAN surveillance system and those with gestational syphilis or toxoplasmosis among children classified as having CZS, based on RESP-Microcefalia records.

Finally, the analysis was restricted to children born to mothers residing in municipalities that reported autochthonous cases of Zika fever in the general population and/or cases of CZS during the study period. The list of municipalities with reported acute Zika virus disease or CZS was obtained from the Brazilian Ministry of Health (DATASUS, TabNet–SINAN) in September 2025.

### Exposure measures

Exposure status was classified into three categories: 1) children with CZS were identified through linkage between live birth records and confirmed or probable CZS cases recorded in RESP, defined according to the Brazilian Ministry of Health surveillance criteria in RESP-Microcefalia^28^; 2) ZIKV-exposed children without CZS were defined as those whose mothers had a confirmed ZIKV infection during pregnancy recorded in SINAN and whose foetus had no corresponding record of confirmed or probable CZS in RESP-Microcefalia. This included children with no RESP notification or with a ruled-out CZS classification; and 3) children were classified as unexposed to ZIKV during pregnancy if their mothers had no reported or confirmed ZIKV infection during pregnancy and if the child was not classified as confirmed or probable CZS in RESP-Microcefalia, regardless of notification status.^34^ No exclusions were made for congenital anomalies unrelated to ZIKV, preserving the population-based structure of the cohort.^34^

### Outcome measures

Our outcome was a combined measure of morbidity and mortality due to community-acquired pneumonia, defined as the first hospitalisation or death with a primary diagnosis or underlying cause of death classified under ICD-10 codes J12–J18. We used the grouped definition because the aetiology of pneumonia is often not established at the time of hospital admission or death, and laboratory confirmation is frequently unavailable or inconclusive, particularly in cases of coinfection or pneumonia with mixed aetiologies.^35^, ^36^, ^37^ Cases of aspiration pneumonitis (ICD-10 code J69) were explicitly excluded from the outcome definition. Although dysphagia and feeding difficulties are well-recognised complications of CZS and represent important risk factors for aspiration events,^20^ aspiration pneumonitis was not included given the distinct pathophysiological mechanism and the risk of outcome misclassification in the context of this aetiological study. Hospitalisation and death were combined into a single composite outcome because the absolute number of deaths was too small to permit separate analyses.

As a negative outcome control, we used hospitalisations and deaths due to external causes, defined as ICD-10 Chapters XX (V01–Y98) and XIX (S00–T98), excluding complications of medical and surgical care (T80–T88). External causes were selected because they are not plausibly related to intrauterine biological mechanisms and, therefore, are not expected to be causally affected by prenatal exposure to ZIKV. Under this assumption, an association between prenatal ZIKV exposure and external causes would suggest the presence of residual confounding or bias rather than a true causal effect. We excluded ICD-10 codes T80–T88, as children with congenital Zika-related conditions tend to have higher healthcare utilisation and more frequent hospital admissions.^11^ This increased exposure to medical procedures may, in turn, elevate the likelihood of procedure-related complications, introducing differential misclassification and violating the assumptions of a valid negative outcome control.

### Covariates

Covariates were selected *a priori* based on literature, and we drew a directed acyclic graph (Supplementary Figure S1 in Supplementary Appendix 1) to inform the analyses. All covariates were measured before or during pregnancy, ensuring that they temporally preceded the outcomes and could not be influenced by postnatal events, thereby minimising the risk of adjustment for mediators or collider bias.

Maternal characteristics included state of residence, maternal age (<20, 20–34, ≥35 years), education (none, ≤7 years, ≥8 years), self-reported race/ethnicity based on skin colour as recorded in the live birth certificate and grouped as White, Black, Mixed (Brown/*Pardo*), and Other (Asian descent and Indigenous), relationship status (with or without partner), adequacy of prenatal care, and year of conception. Adequate prenatal care was defined according to the Brazilian Ministry of Health recommendations regarding the minimum number and timing of prenatal visits. Based on gestational age–specific cut-offs, prenatal care was considered adequate if the mother attended at least two visits by 20 weeks, three by 27 weeks, four by 30 weeks, five by 33 weeks, and six visits by delivery.^34^

Household socioeconomic conditions were obtained from the CadUnico database and included household location (urban/rural), construction material (masonry/brick, coated or uncoated taipa, wood, other), water supply (public system, well, or other), waste disposal/garbage collection, and sanitation type. Seasonal variation was accounted for using trimester of conception, to capture temporal fluctuations in ZIKV transmission and respiratory infection risk. Individual-level data on some postnatal determinants of community-acquired pneumonia, including breastfeeding duration and exposure to secondhand tobacco smoke, were not captured in the administrative databases underlying the CIDACS Birth Cohort and were therefore unavailable.

### Statistical analysis

Baseline characteristics were summarised by exposure group, with continuous variables described using medians and interquartile ranges (IQRs) and categorical variables as absolute and relative frequencies.

To describe absolute risk over time in the presence of competing events, cumulative incidence functions (CIFs) for pneumonia-related morbidity and mortality were estimated using the Aalen–Johansen estimator, accounting for deaths from other causes as a competing risk and for the variable follow-up time across the cohort, which arose from the 2015–2018 birth window; estimates at later time points are therefore based on progressively fewer children still under observation. CIFs were used to characterise risk trajectories across exposure groups, and differences between curves were assessed using Grey's test.

Our target estimand was the total effect of prenatal ZIKV exposure on pneumonia-related morbidity and mortality, defined *a priori* and interpreted under standard identifiability assumptions, as encoded in the directed acyclic graph (Supplementary Figure S1 in Supplementary Appendix 1). The three analytical approaches were selected to probe this estimand under different and largely non-overlapping assumptions.

Subsequent analyses estimated this effect using the three approaches described below. All analyses were conducted using R (R Foundation for Statistical Computing, Vienna, Austria), with the packages *WeightIt*, *cobalt*, *survival*, *MatchIt*, *broom*, and *tidyverse*.

#### Multivariable regression analyses

In these analyses we used inverse probability of treatment weights (IPTW) derived from a generalised boosted model to calculate the probability of being exposed to ZIKV during pregnancy.^38^ Separate weights were calculated separately for CZS versus unexposed and ZIKV-exposed without CZS versus unexposed using the baseline covariates (Supplementary Figure S1). Stabilised IPTW weights were generated and truncated at the 1st and 99th percentiles to limit the influence of extreme values. Covariate balance between exposure groups was assessed using standardised mean differences, with absolute values below 0.10 indicating adequate balance. Weighted cause-specific Cox proportional hazards models were then fitted using the trimmed treatment effect on the treated weights. Hazard ratios and 95% CI were reported. Proportional hazards assumptions were evaluated using Schoenfeld residuals. Missing data in the covariates was addressed in the main analysis by utilising a missing indicator.^39^

We estimated cause-specific hazards rather than subdistribution (Fine–Grey) hazards because our aim was aetiological: cause-specific hazards quantify the effect of an exposure on the event rate among those still at risk, whereas Fine–Grey models target absolute risk and are better suited to prediction.^40^^,^^41^

We conducted a series of sensitivity analyses to assess the robustness of the estimated associations derived from the IPTW. First, the same IPTW-weighted cause-specific Cox proportional hazards modelling framework was applied after restricting the sample to term births (≥37 weeks’ gestation) and infants without low birthweight (≥2500 g), to evaluate whether the observed associations persisted. Second, the primary IPTW cause-specific Cox analysis was repeated in a restricted sample of children born to mothers with laboratory-confirmed ZIKV infection, to examine the robustness of the findings to potential exposure misclassification. Third, to evaluate the robustness of the findings to an alternative confounding-control strategy and to reduce reliance on model-based adjustment, we implemented a 1:4 matched cohort design comparing ZIKV-exposed (both CZS and non-CZS) and unexposed children. Exact matching was applied on maternal characteristics (state of residence, maternal age, education, race/ethnicity, relationship status, adequacy of prenatal care, and year and trimester of conception), and propensity score matching was performed on household-level socioeconomic characteristics. In the matched cohort, cause-specific Cox proportional hazards models were fitted, stratified by matched sets and with robust standard errors clustered by matched set. Covariates used in the matching process that remained imbalanced after matching (standardised mean difference >0.1) were additionally included in the models.

#### Within-sibling comparison

We conducted a within-sibling comparison to address potential unmeasured familial and genetic confounding shared among siblings. This analysis was restricted to mothers contributing more than one live birth during the study period (2015–2018), with discordant exposure status among their children. We fitted Cox proportional hazards models with robust standard errors clustered at the maternal level, adjusting for maternal age using cubic splines and for calendar time using year-by-region fixed effects. Birth order was not included as an adjustment variable in this analysis. This decision reflected the highly concentrated distribution of events: 92.3% of pneumonia-related morbidity and mortality events occurred in CZS children, of whom 88.4% were second-born; consequently, 94% of all outcome events were observed among children of birth order two. Including birth order as a covariate under these conditions would have introduced minimal variability, increasing the risk of model instability and over-adjustment.

#### Negative outcome control analysis

We performed a negative outcome control to assess whether the associations observed for pneumonia-related morbidity or mortality could be explained by residual confounding or systematic bias. The same IPTW cause-specific Cox modelling framework used in the multivariable regression analyses was applied.

#### Use of generative artificial intelligence

The analysis code, written in R, was developed entirely by the authors without the support of generative artificial intelligence. All analytical decisions, including the definition of the analytical methods, the selection of covariates, the definition of the outcome, and the interpretation of the results, were made solely by the researchers, without the use of artificial intelligence. A generative AI tool (Claude, Anthropic) was used only when an error message occurred during code execution that the researchers had not been able to resolve through their own initial attempts, for example, functions deprecated following package updates or date-class (IDate) incompatibilities that prevented data merging. In such cases, the error message was submitted to the tool to better understand the cause of the error and possible corrections. Whenever this occurred, the researchers validated each correction by running the code and confirming that the results reproduced the expected values. The authors take full responsibility for the analysis and its outputs.

### Ethics statement

This work is part of the Study of Early-Life Exposures During Developmental Stages (SEEDS) and was approved by the Research Ethics Committee (CEP) of the Gonçalo Moniz Research Centre (CAAE: 71054923.8.0000.0040) and by the London School of Hygiene & Tropical Medicine Ethics Committee (Ref.: 29560). Informed consent was waived because we are using administrative collected de-identified data and analysed under strict data security procedures at CIDACS,^32^ in accordance with the Brazilian General Data Protection Law.^33^

### Role of funding source

The funders of the study had no role in study design, data collection, data analysis, data interpretation, or writing of the report.

## Results

From 2015 to November 2018, the CIDACS Birth Cohort comprised 5,695,179 new live births (Fig. 1). After applying the study eligibility criteria, the final study population comprised 4,108,409 unexposed children, 3900 children ZIKV-exposed without CZS, and 1786 children with CZS.

**Fig. 1:**
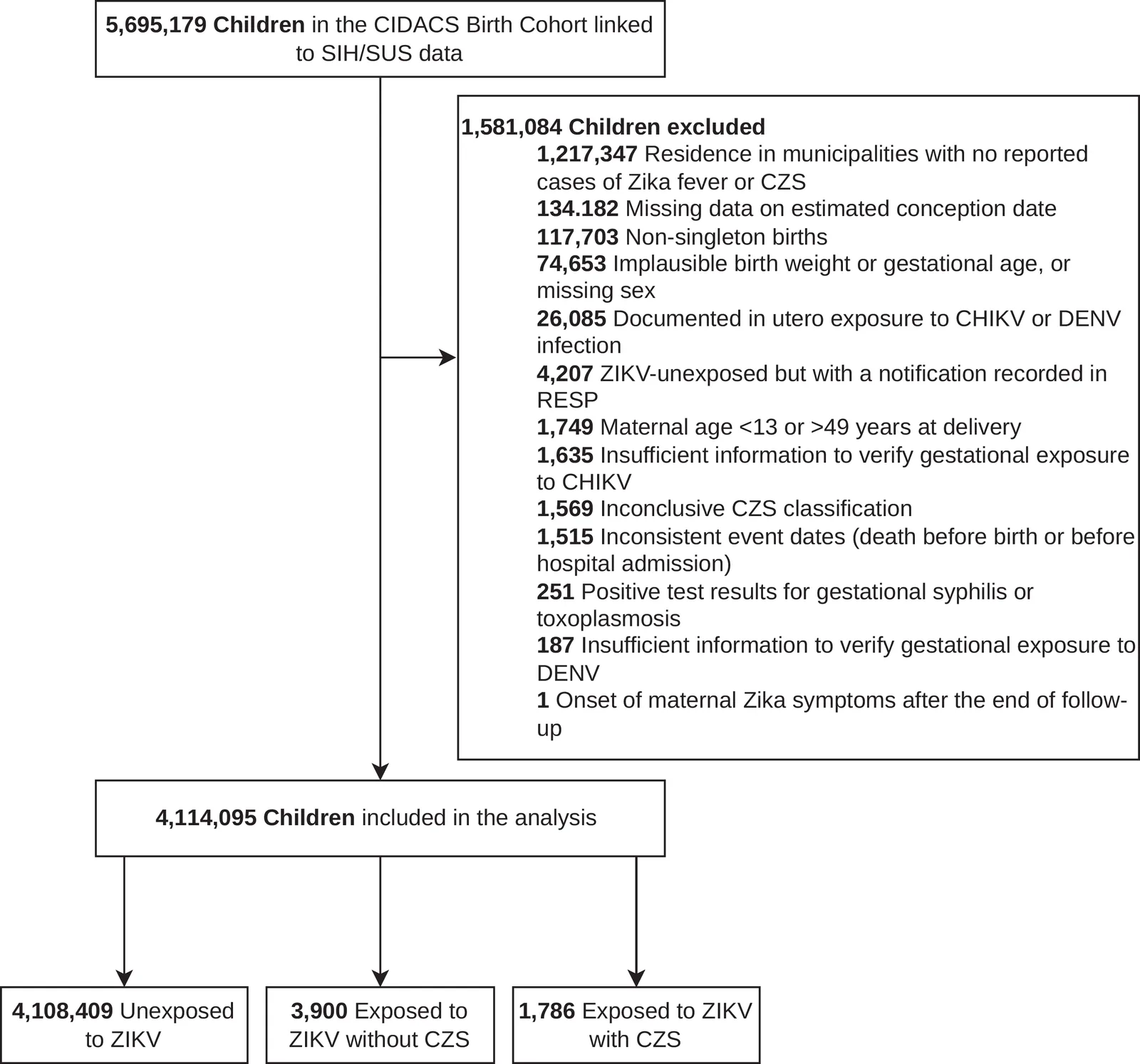
**Flowchart of the study population.** The cohort comprises live births from January 1, 2015, to November 30, 2018, to mothers registered in CadÚnico, drawn from the CIDACS Birth Cohort and linked to SIH/SUS hospitalisation data. Exclusion criteria were applied sequentially, so that each child is counted only once, under the first criterion met. Children with more than one hospital admission contributed a single record, retaining the first admission; this step reduced the number of records but not the number of children. CadUnico, Unified Registry for Social Programs; CHIKV, chikungunya virus; CIDACS, Centre for Data and Knowledge Integration for Health; CZS, congenital Zika syndrome; DENV, dengue virus; RESP, Public Health Events Registry; SIH/SUS, Hospital Information System of the Brazilian Unified Health System; ZIKV, Zika virus.

Children with CZS differed markedly from the other two groups across perinatal and socioeconomic characteristics (Table 1). They were more likely to be born with low birth weight (613 [34.3%] below 2500 g, compared with approximately 299 [7.7%] of ZIKV-exposed without CZS and 305,028 [7.4%] of unexposed children) and preterm (347 [19.4%] before 37 weeks, versus 391 [10.0%] and 429,054 [10.5%], respectively). Their births were concentrated in the Northeast region (1186; 66.4%), and they were more likely to live in households with less adequate water supply, sanitation, and waste disposal. In contrast, ZIKV-exposed children without CZS were broadly similar to unexposed children. Pneumonia-related hospitalisation was recorded in 253 (14.2%) children with CZS, compared with 141 (3.6%) ZIKV-exposed children without CZS and 144,340 (3.5%) unexposed children. Pneumonia-related deaths were rare in all groups: 3 (0.2%) among children with CZS, 1 (<0.1%) among ZIKV-exposed children without CZS, and 1679 (<0.1%) among unexposed children. All-cause mortality followed a similar pattern, occurring in 178 (10.0%) children with CZS, 36 (0.9%) ZIKV-exposed children without CZS, and 44,799 (1.1%) unexposed children.

**Table 1:** Baseline maternal, perinatal, and household characteristics of the study population according to prenatal ZIKV exposure status.

Characteristic	ZIKV-unexposed n = 4,108,409^a^	ZIKV-exposed (non-CZS)n = 3,900^a^	ZIKV-exposed CZS n = 1,786^a^
Sex			
Male	2,105,098 (51.2%)	2080 (53.3%)	838 (46.9%)
Female	2,003,311 (48.8%)	1820 (46.7%)	948 (53.1%)
Birth weight (g)			
Median (Q1, Q3)	3230 (2922; 3540)	3231 (2915; 3556)	2710 (2320; 3040)
Birth weight (g)			
<1500	42,633 (1.0%)	30 (0.8%)	74 (4.1%)
1500–2499	262,395 (6.4%)	269 (6.9%)	539 (30.2%)
≥2500	3,803,381 (92.6%)	3601 (92.3%)	1173 (65.7%)
Gestational age at birth (weeks)			
Median (Q1, Q3)	39 (38; 40)	39 (38; 40)	39 (37; 40)
Gestational age at birth (weeks)			
<32	56,474 (1.4%)	32 (0.8%)	47 (2.6%)
32–36	372,580 (9.1%)	359 (9.2%)	300 (16.8%)
≥37	3,679,355 (89.6%)	3509 (90.0%)	1439 (80.6%)
Missing	0 (0.0%)	0 (0.0%)	0 (0.0%)
Birth year			
2015	1,336,305 (32.5%)	49 (1.3%)	811 (45.4%)
2016	947,165 (23.1%)	3423 (87.8%)	742 (41.5%)
2017	953,141 (23.2%)	295 (7.6%)	160 (9.0%)
2018	871,798 (21.2%)	133 (3.4%)	73 (4.1%)
Missing	0 (0.0%)	0 (0.0%)	0 (0.0%)
Mode of delivery			
Vaginal	2,160,142 (52.6%)	1916 (49.1%)	940 (52.6%)
Cesarean	1,945,930 (47.4%)	1983 (50.8%)	845 (47.3%)
Missing	2337 (0.1%)	1 (<0.1%)	1 (0.1%)
Prenatal appointments			
Inadequate	912,172 (22.2%)	572 (14.7%)	468 (26.2%)
Adequate	3,101,748 (75.5%)	3278 (84.1%)	1266 (70.9%)
Missing	94,489 (2.3%)	50 (1.3%)	52 (2.9%)
Mother age (years)			
Median (Q1, Q3)	24 (20; 30)	25 (20; 30)	24 (19; 29)
Mother age (years)			
<20	866,404 (21.1%)	750 (19.2%)	456 (25.5%)
20–34	2,825,430 (68.8%)	2717 (69.7%)	1159 (64.9%)
≥35	416,575 (10.1%)	433 (11.1%)	171 (9.6%)
Missing	0 (0.0%)	0 (0.0%)	0 (0.0%)
Maternal race/ethnicity			
White	952,335 (23.2%)	878 (22.5%)	261 (14.6%)
Black	270,803 (6.6%)	217 (5.6%)	114 (6.4%)
Asian descendant	11,777 (0.3%)	8 (0.2%)	4 (0.2%)
Brown/*Pardo*	2,653,838 (64.6%)	2643 (67.8%)	1293 (72.4%)
Indigenous	31,434 (0.8%)	3 (0.1%)	10 (0.6%)
Missing	188,222 (4.6%)	151 (3.9%)	104 (5.8%)
Marital status			
Single	2,138,647 (52.2%)	2505 (64.4%)	931 (52.3%)
Married	861,991 (21.1%)	727 (18.7%)	360 (20.2%)
Widowed	6245 (0.2%)	5 (0.1%)	3 (0.2%)
Divorced	41,769 (1.0%)	43 (1.1%)	12 (0.7%)
Civil union	1,018,954 (24.9%)	586 (15.1%)	459 (25.8%)
Missing	26,041 (0.6%)	24 (0.6%)	15 (0.8%)
Educational attainment (years)			
0–3	113,067 (2.8%)	70 (1.8%)	60 (3.4%)
4–7	866,626 (21.4%)	692 (18.0%)	433 (24.6%)
8–11	2,727,438 (67.2%)	2715 (70.4%)	1124 (63.9%)
≥12	328,727 (8.1%)	374 (9.7%)	132 (7.5%)
Missing	19,878 (0.5%)	4 (0.1%)	11 (0.6%)
Geographic region of Brazil			
Southeast	1,483,706 (36.1%)	2281 (58.5%)	369 (20.7%)
South	342,665 (8.3%)	8 (0.2%)	24 (1.3%)
Central West	346,714 (8.4%)	667 (17.1%)	120 (6.7%)
Northeast	1,441,745 (35.1%)	587 (15.1%)	1186 (66.4%)
North	493,579 (12.0%)	357 (9.2%)	87 (4.9%)
Missing	0 (0.0%)	0 (0.0%)	0 (0.0%)
Pneumonia (hospitalization)			
No	3,964,069 (96.5%)	3759 (96.4%)	1533 (85.8%)
Yes	144,340 (3.5%)	141 (3.6%)	253 (14.2%)
Pneumonia (deaths)			
No	4,106,730 (100.0%)	3889 (100.0%)	1783 (99.8%)
Yes	1679 (<0.1%)	1 (<0.1%)	3 (0.2%)
Death (all-causes)			
No	4,063,610 (98.9%)	3864 (99.1%)	1608 (90.0%)
Yes	44,799 (1.1%)	36 (0.9%)	178 (10.0%)
External causes (negative outcome)			
No	4,089,128 (99.5%)	3874 (99.3%)	1776 (99.4%)
Yes (hospitalization)	17,711 (0.4%)	23 (0.6%)	10 (0.6%)
Yes (death)	1229 (<0.1%)	3 (0.1%)	0 (0.0%)
Timing of exposure to Zika during pregnancy (weeks)			
Median (Q1, Q3)	–	23.6 (16.0; 30.6)	17.5 (10.0; 34.7)
Trimester of maternal Zika infection			
First	–	655 (16.8%)	89 (5.0%)
Second	–	1634 (41.9%)	56 (3.1%)
Third	–	1611 (41.3%)	93 (5.2%)
Missing	–		1548 (86.7%)
Location			
Urban	3,235,579 (78.8%)	3512 (90.1%)	1296 (72.6%)
Rural	752,297 (18.3%)	260 (6.7%)	436 (24.4%)
Missing	120,533 (2.9%)	128 (3.3%)	54 (3.0%)
Household water supply			
Public network	2,878,614 (70.1%)	3086 (79.1%)	1157 (64.8%)
Well/Spring	726,042 (17.7%)	427 (10.9%)	357 (20.0%)
Other	314,981 (7.7%)	189 (4.8%)	197 (11.0%)
Missing	188,772 (4.6%)	198 (5.1%)	75 (4.2%)
Household waste disposal			
Collected	3,096,575 (75.4%)	3353 (86.0%)	1264 (70.8%)
Burned/Buried	561,750 (13.7%)	214 (5.5%)	283 (15.8%)
Open-air dump	216,619 (5.3%)	97 (2.5%)	143 (8.0%)
Other	44,669 (1.1%)	38 (1.0%)	21 (1.2%)
Missing	188,796 (4.6%)	198 (5.1%)	75 (4.2%)
Household sewage system			
Public sewer network	1,898,212 (46.2%)	2366 (60.7%)	725 (40.6%)
Septic tank	544,361 (13.2%)	390 (10.0%)	295 (16.5%)
Rudimentary cesspit	897,352 (21.8%)	624 (16.0%)	389 (21.8%)
Ditch	465,289 (11.3%)	241 (6.2%)	249 (13.9%)
Other	67,674 (1.6%)	58 (1.5%)	32 (1.8%)
Missing	235,521 (5.7%)	221 (5.7%)	96 (5.4%)
Housing construction material			
Masonry	3,028,782 (73.7%)	3283 (84.2%)	1416 (79.3%)
Covered wattle and daub	104,942 (2.6%)	36 (0.9%)	66 (3.7%)
Uncovered wattle and daub	104,858 (2.6%)	28 (0.7%)	60 (3.4%)
Wood	457,869 (11.1%)	246 (6.3%)	67 (3.8%)
Other	223,126 (5.4%)	109 (2.8%)	102 (5.7%)
Missing	188,832 (4.6%)	198 (5.1%)	75 (4.2%)

CZS, Congenital Zika Syndrome; ZIKV, Zika vírus.

a n (%) for categorical variables; median (Q1, Q3) for continuous variables.

Children with CZS experienced a higher risk of pneumonia-related morbidity and mortality throughout follow-up (Fig. 2). By 42 months of age, the cumulative incidence of pneumonia-related hospitalisation or death reached 15.6% (95% CI 13.7–17.5) among children with CZS, compared with 3.9% (95% CI 3.2–4.7) among ZIKV-exposed children without CZS and 4.6% (95% CI 4.6–4.7) among unexposed children. Notably, the cumulative incidence function revealed an early steep rise of approximately 2% in the CZS group within the first weeks of life. In contrast, CIF curves for children exposed to ZIKV in utero without CZS closely overlapped with those of unexposed children. Grey's test provided evidence of differences in cumulative incidence between children with CZS and unexposed children (p < 0.001), whereas no evidence of differences was observed between ZIKV-exposed children without CZS and unexposed children (p = 0.790).

**Fig. 2:**
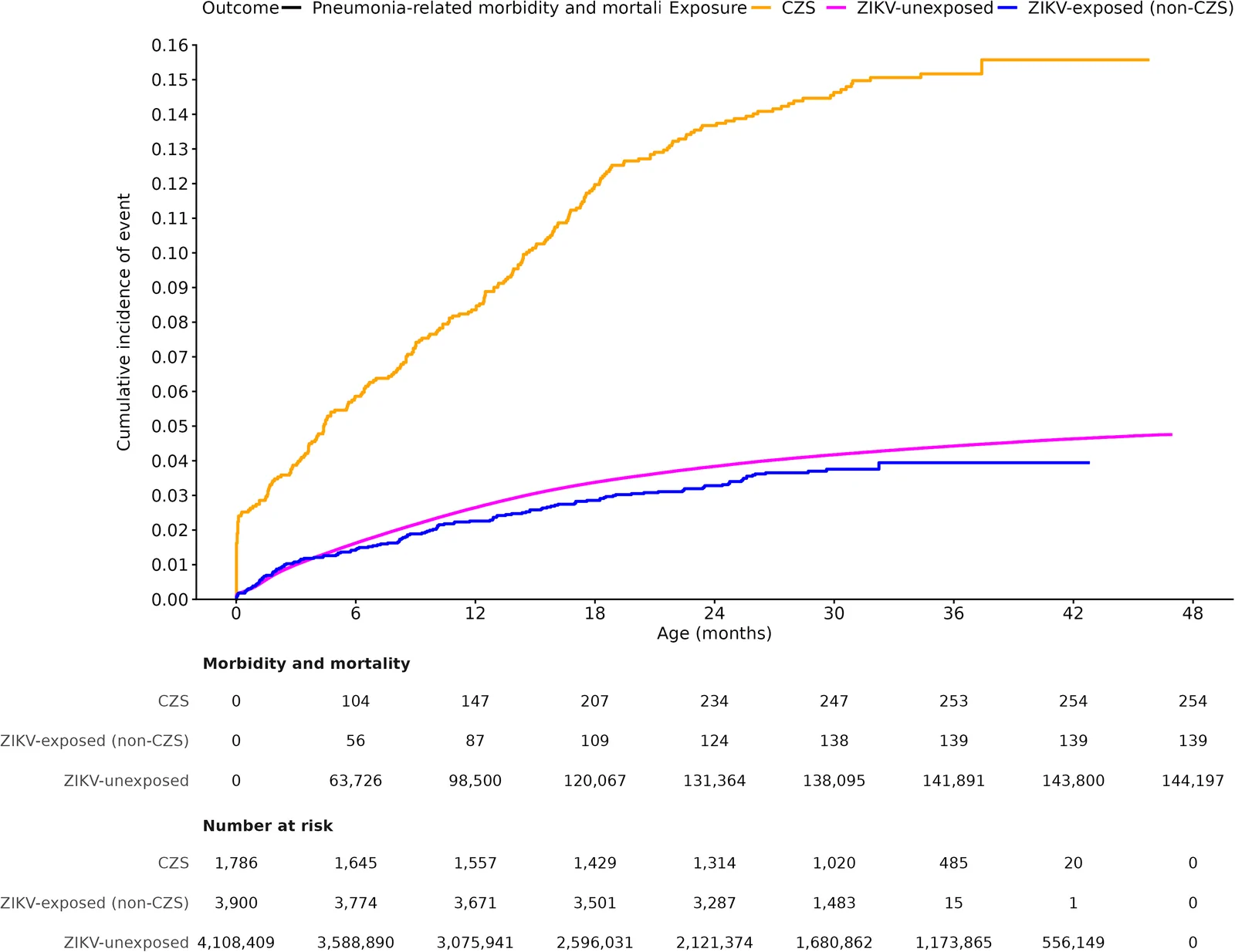
**Cumulative incidence of pneumonia-related morbidity and mortality by antenatal ZIKV exposure category.** Cumulative incidence was estimated using the Aalen–Johansen estimator, accounting for the competing risk of death from other causes and for the variable follow-up time across the cohort arising from the 2015–2018 birth window. Cumulative incidence values reported in the text refer to 42 months of age, the latest time point at which sufficient children remained under observation. ZIKV, Zika virus; CZS, Congenital Zika Syndrome.

Effect estimates from IPTW-weighted multivariable regression models, within-sibling comparisons, and negative outcome control analyses are presented in Fig. 3. Children with CZS consistently showed a substantially higher risk of pneumonia-related morbidity and mortality compared with unexposed children. In the IPTW-adjusted model, CZS was associated with a more than fourfold increased risk of pneumonia-related hospitalisation or death compared with unexposed children (aHR: 4.12, 95% CI: 3.64–4.66). The point estimate from the within-sibling comparison (aHR: 4.15, 95% CI: 1.62–10.64) was closely consistent with that from the IPTW-adjusted model, although its confidence interval was considerably wider. This wider interval reflects the limited number of siblings discordant for exposure and outcome available for this analysis (n = 256), which reduces precision but not the consistency of the estimated association. In the negative outcome control analysis, using hospitalisation or death due to external causes as the outcome, the association for CZS was close to the null (aHR: 1.08, 95% CI: 0.58–2.02).

**Fig. 3:**
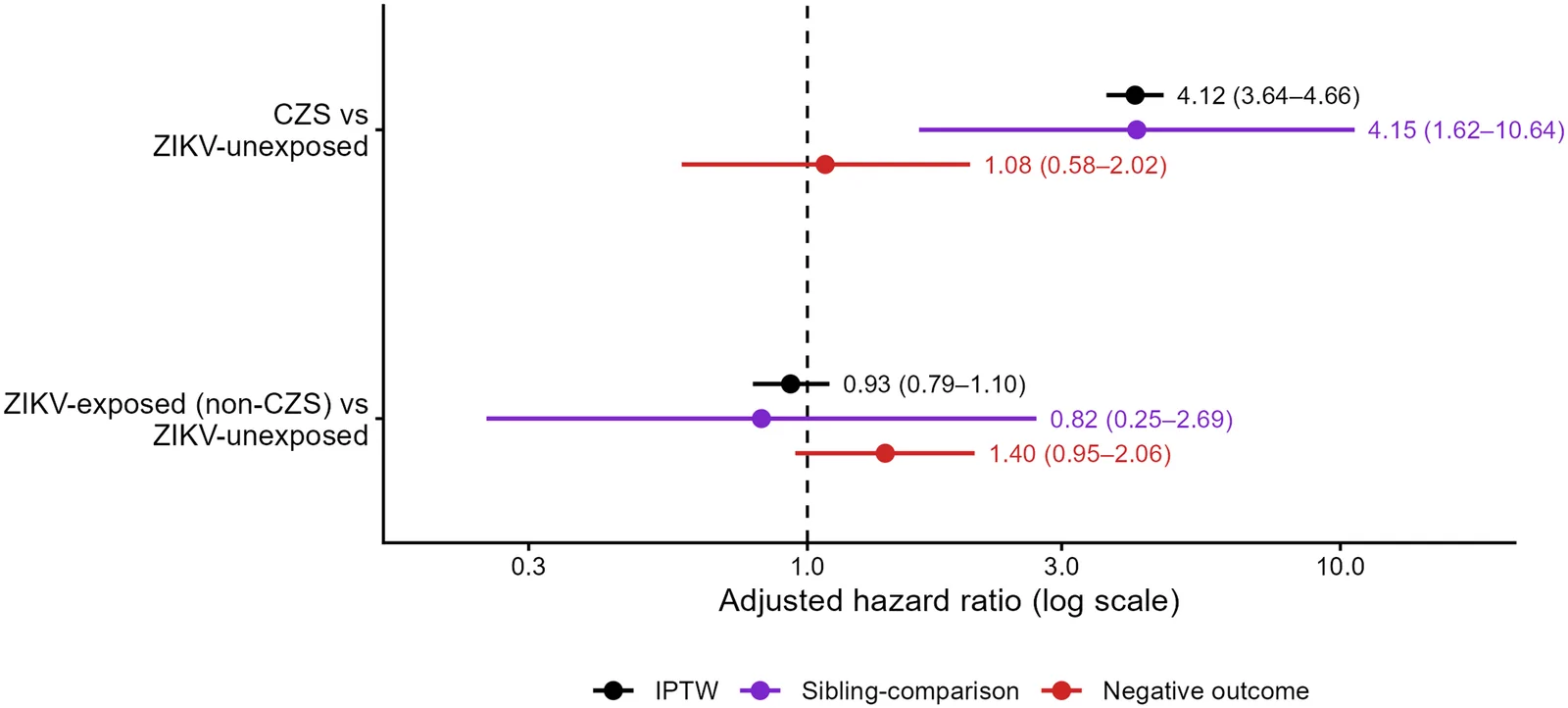
**Adjusted hazard ratios for pneumonia-related morbidity and mortality across triangulated analytical approaches (IPTW-adjusted model, n = 4,108,409; sibling comparison, n = 256; negative control outcome, n = 4,108,409).** ZIKV, Zika virus; CZS, Congenital Zika Syndrome; IPTW, Inverse Probability of Treatment Weighting.

Children exposed to ZIKV in utero without CZS did not show evidence of an increased risk of pneumonia-related morbidity and mortality compared with unexposed children. In the multivariable regression models, no association was observed between prenatal ZIKV exposure without CZS and pneumonia-related hospitalisation or death (aHR: 0.93, 95% CI: 0.79–1.10), similarly, the within-sibling comparison showed no evidence of an increased risk among ZIKV-exposed children without CZS relative to their unexposed siblings (HR: 0.82, 95% CI: 0.25–2.69). Consistent with these findings, the negative outcome control analysis showed no association for ZIKV-exposed children without CZS compared with unexposed children (aHR: 1.40, 95% CI: 0.95–2.06).

The Supplementary Appendix 1 includes covariate balance diagnostics for the IPTW analyses of both the primary outcome and the negative control outcome (Supplementary Figures S2 and S3; Supplementary Tables S1–S4 in Supplementary Appendix 1), as well as descriptive comparisons of maternal characteristics for those included and excluded from the sibling analysis (Supplementary Tables S5 and S6 in Supplementary Appendix 1).

### Sensitivity analyses

For the comparison between children with CZS and ZIKV-unexposed children, effect estimates remained consistent across sensitivity analyses. Similar associations were observed when restricting the sample to term births with normal birthweight (aHR: 4.33, 95% CI: 3.70–5.07), to children born to mothers with laboratory-confirmed ZIKV infection (aHR: 4.26, 95% CI: 3.73–4.87), and when using a 1:4 matched cohort design (aHR: 4.33, 95% CI: 3.61–5.19). Likewise, for children exposed to ZIKV in utero without CZS, findings were consistent across sensitivity analyses (Fig. 4) (Supplementary Figures S4–S7; Supplementary Tables S7–S18 in Supplementary Appendix 1).

**Fig. 4:**
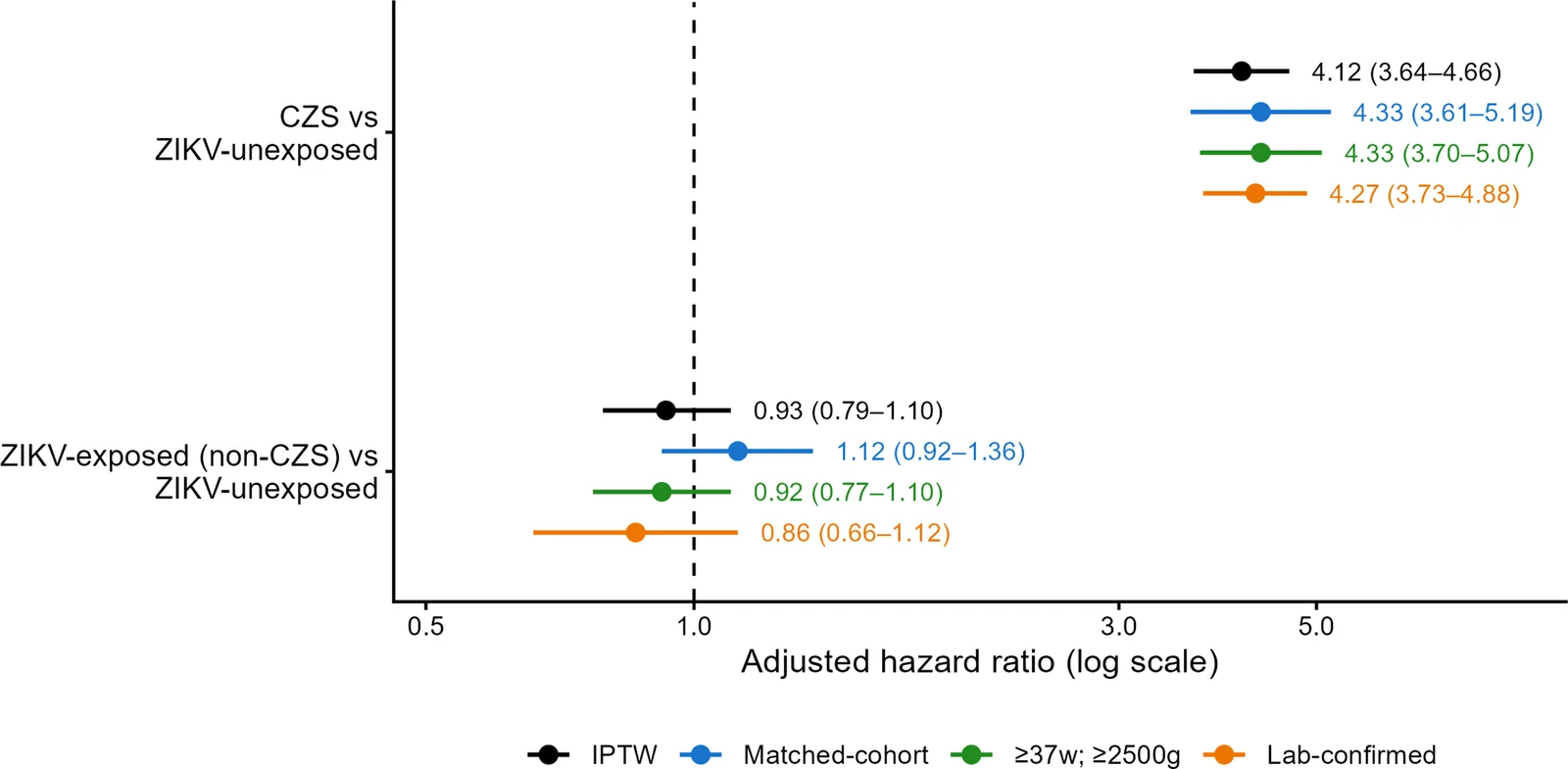
**Adjusted hazard ratios for pneumonia-related morbidity and mortality following prenatal Zika virus exposure, including the multivariable regression analyses and three sensitivity analyses.** ZIKV, Zika virus; CZS, Congenital Zika Syndrome; IPTW, Inverse Probability of Treatment Weighting.

## Discussion

In this nationwide, registry-based study conducted in Brazil, prenatal ZIKV exposure was associated with a more than fourfold higher risk of pneumonia-related morbidity and mortality among children with CZS, whereas exposed children without CZS showed no increased risk. By 42 months, an estimated 15% of children with CZS had been hospitalised for or died from community-acquired pneumonia, compared with 3–4% of children in the other two groups. This difference in absolute risk, of a magnitude of roughly one in seven affected children, underscores the clinical and public health relevance of the finding. The validity and robustness of these findings are supported by the lack of association observed in the negative outcome control analysis and by the consistency of results across multiple analyses. These findings extend existing evidence on the long-term consequences of maternal infections,^42^^,^^43^ and suggest that the respiratory burden associated with prenatal ZIKV exposure is primarily concentrated among children with congenital manifestations.

The absence of an increased risk of severe pneumonia among children exposed to ZIKV in utero who did not develop CZS is a reassuring finding, particularly as the vast majority of prenatally exposed children do not present with congenital manifestations.^17^, ^18^, ^19^, ^20^ However, it should be interpreted as specific to severe, hospitalised pneumonia: children exposed to ZIKV in utero who do not develop CZS have been shown to experience other adverse outcomes, including neurodevelopmental delays in language, cognitive, and motor domains.^17^^,^^18^ Furthermore, given that diagnostic test availability limitations may have introduced some ZIKV exposure misclassification, continued clinical and epidemiological surveillance of the entire exposed cohort remains prudent.

The excess pneumonia-related morbidity and mortality among children with CZS is likely to arise through more than one mechanism. The most direct is neurological: severe brain injury frequently results in oropharyngeal dysphagia, recurrent aspiration, impaired airway clearance, and an ineffective cough, which together predispose to lower respiratory tract infection and are well recognised as a leading cause of respiratory morbidity and mortality in children with severe neurodisability.^44^^,^^45^ Children with CZS commonly present with dysphagia and feeding difficulties,^20^ placing them at particular risk through this pathway. The marked neurotropism of ZIKV^46^^,^^47^ may also disrupt central neural pathways involved in respiratory control, predisposing affected children to airway dysfunction.

Beyond these neurological mechanisms, congenital ZIKV infection may act through immune and inflammatory pathways that are distinct from, and may add to, the aspiration-related mechanism described earlier. ZIKV infection during foetal development may induce an exaggerated inflammatory state, leading to long-term immune dysregulation and increased vulnerability to infectious conditions in childhood,^48^ and it has been shown to interfere with pluripotent stem cell differentiation and organogenesis, with potential implications for pulmonary development.^49^ Virus-induced epigenetic modifications during gestation may further affect genes related to immune regulation and lung development, increasing susceptibility to respiratory and infectious diseases later in life.^50^, ^51^, ^52^ Consistent with an immune-mediated pathway, ZIKV-exposed children who have normal clinical findings at birth have been shown to exhibit sustained low-grade inflammation, Th1-skewed immune profiles, and impaired humoural responses to routine childhood vaccinations, particularly those dependent on Th2-mediated immunity such as DTaP.^15^ That such alterations can arise in the absence of overt structural injury suggests that this immune-mediated susceptibility may operate independently of the swallowing impairments that predispose to aspiration. Why these alterations do not translate into a detectable excess of severe pneumonia among ZIKV-exposed children without CZS in our cohort remains unclear and merits further investigation.

Prenatal exposure to other congenital infections (grouped under the STORCH acronym) has been associated with an increased risk of adverse respiratory outcomes in childhood, although the magnitude and frequency of these effects vary across pathogens. Severe pulmonary complications and/or increased hospitalisation rates have been described in children with congenital rubella,^53^ cytomegalovirus,^54^^,^^55^ and syphilis.^56^ These risks are markedly elevated in children with established congenital disease,^57^^,^^58^ underscoring the relevance of structural injury, immune dysregulation, or both in mediating susceptibility to severe infections.^57^, ^58^, ^59^, ^60^ In this context, the concentration of excess pneumonia-related morbidity and mortality among children with CZS observed in our study, coupled with the absence of increased risk among ZIKV-exposed children without congenital manifestations, aligns with patterns described for other congenital infections.

While symptomatic maternal dengue and chikungunya infections during pregnancy have been associated with adverse perinatal outcomes, and in-utero chikungunya exposure has been linked to increased all-cause hospitalisation in early childhood,^61^^,^^62^ the existing literature has not identified respiratory disease as a prominent outcome of prenatal exposure to either virus. This divergence suggests that the excess pneumonia-related morbidity and mortality observed in our cohort is likely specific to the unique pathogenic profile of congenital infection rather than a general consequence of prenatal arboviral exposure. However, this hypothesis warrants direct, comparative investigation in cohorts of children exposed in utero to other emerging arboviruses.

This study has several strengths. We used a large, nationwide, population-based birth cohort derived from linked administrative health and social registries in Brazil, enabling the evaluation of severe pneumonia outcomes requiring hospitalisation or resulting in death. A major strength of this study is the use of a triangulation framework integrating complementary analytical approaches with distinct assumptions and sources of bias. We combined population-level analyses using IPTW-weighted regression, which account for measured confounding, with within-sibling comparisons that control for shared familial, genetic, and early-life environmental factors, thereby reducing bias from unmeasured confounding at the family level. Finally, we applied a negative outcome control analysis to assess the extent of residual confounding. The consistency of findings across these approaches, each subject to different potential biases, strengthens confidence in the observed associations and reduces reliance on any single method.

Several limitations should be acknowledged. Prenatal ZIKV exposure was identified through passive surveillance and is likely underascertained, particularly for asymptomatic maternal infections and for infections occurring early in the epidemic, before Zika diagnostic testing was available; this may have resulted in nondifferential misclassification and bias toward the null. Children with CZS are more likely to be captured by surveillance systems because the condition presents with clear clinical manifestations at birth, whereas prenatal ZIKV exposure without congenital manifestations may remain undetected, particularly given the high proportion of asymptomatic infections in adults.^63^^,^^64^ In addition, selection bias is possible in the group of children exposed to ZIKV in utero who did not develop CZS, as their mothers had higher socioeconomic status and may therefore have had greater access to outpatient care that did not require hospitalisation. Outcomes were defined using hospitalisation and mortality records based on ICD-10 codes, capturing severe pneumonia but not milder infections managed in outpatient settings. Although aspiration pneumonitis (ICD-10 code J69) was excluded from the outcome definition (as detailed in the Methods), some episodes of aspiration-related pneumonia may nonetheless have been misclassified at hospital admission as community-acquired pneumonia. This potential misclassification is particularly relevant among children with CZS, given their predisposition to aspiration events.^20^ Beyond aspiration-related misclassification, the accuracy of ICD-10 coding in the SIH/SUS is subject to well-documented limitations, as the system was designed primarily for reimbursement and coding quality is affected by operational factors such as limited staff training and turnover.^65^ These limitations may be more pronounced where access to diagnostic testing is more constrained, as in some peripheral areas of Brazil. Because our outcome was defined by the aggregated J12–J18 group rather than by aetiology-specific codes, misclassification among individual codes within this group does not affect outcome ascertainment, and any residual misclassification into or out of the J12–J18 group would most plausibly be nondifferential with respect to prenatal ZIKV exposure, biasing estimates toward the null rather than accounting for the associations observed. A further consideration is the potential for surveillance (ascertainment) bias in outcome detection. Children with CZS undergo more intensive medical follow-up and have more frequent health-system contact than unaffected children,^11^ which could increase the likelihood that pneumonia is detected and recorded, biasing the CZS estimate away from the null. The magnitude of this bias cannot be precisely quantified with the available data. However, because our outcome captures only severe disease (hospitalisation or death), which reflects clinical severity rather than frequency of health-system contact, and because our negative outcome control showed no association in children with CZS, differential ascertainment is unlikely to account for the observed increase in risk.

Due to the administrative nature of our dataset, certain variables such as breastfeeding duration and passive smoke exposure were not available. Because these factors act primarily as mediators rather than confounders in the pathway between prenatal ZIKV exposure and pneumonia, adjusting for them would have risked overadjustment bias. We acknowledge, however, that breastfeeding and household smoke exposure may not act solely as mediators, as they are also shaped by maternal and socioeconomic characteristics and could therefore introduce residual confounding. Much of this is likely captured by the socioeconomic variables already included in our IPTW model, and the null result of our negative outcome control analysis argues against substantial residual confounding. Finally, because the CIDACS Birth Cohort is built from the CadUnico registry, it predominantly represents socially vulnerable, lower-income families rather than the entire Brazilian population. However, this population bears a disproportionate share of both the ZIKV and severe respiratory disease burdens. Because these families rely almost exclusively on the SUS, this reliance limits the under-ascertainment of severe clinical outcomes within our cohort. Consequently, while caution is warranted when extrapolating these findings to higher-income populations, our results apply directly to the very group where the public health impact is greatest. Nonetheless, it remains highly plausible that CZS increases the risk of community-acquired pneumonia in other socioeconomic groups as well.

In conclusion, most children exposed to ZIKV in utero do not develop CZS, and for these children ZIKV exposure was not associated with an increased risk of pneumonia-related morbidity and mortality, a reassuring finding for the large majority of exposed children. In contrast, children with CZS exhibited a markedly increased risk. Our results underscore the importance of long-term clinical follow-up and targeted respiratory prevention strategies for children with CZS. Whether specific measures such as immunisation against respiratory pathogens can mitigate this excess risk warrants dedicated evaluation, and our findings provide a rationale for prioritising this high-risk group in future prevention efforts.

## Contributors

EFO and ESP conceptualised the study and wrote the manuscript's first draft. MLB secured data access. ESP and TC-S supervised the data analysis. EFO, ESP, and TC-S defined the methodology. EFO conducted the formal analysis. EFO, TC-S, JGGT, JVBB, BHU, EBB, MGT, MLB, and ESP contributed to the writing, reviewing, and editing of the manuscript. EFO and ESP decided to submit the manuscript for publication. EFO, ESP, and TC-S accessed and verified the raw data in the study. All authors read and approved the final version.

## Data sharing statement

The relevant data are available in the manuscript and the appendix. Data that are not presented in the Article or appendix are available upon reasonable request to CIDACS.

Any person who wishes to receive authorisation must: (i) be affiliated to CIDACS or be accepted as collaborators; (ii) present a detailed research project together with approval by an appropriate Brazilian institutional research ethical committee; (iii) provide a clear data plan restricted to the objectives of the proposed study and a summary of the analyses plan intended to guide the linkage and or data extraction of the relevant set of records and variables; (iv) sign terms of responsibility regarding the access and use of data; and (v) perform the analyses of datasets provided using the CIDACS data environment, a safe and secure infrastructure that provides remote access to de-identified or anonymised datasets and analysis tools.

## Declaration of interests

ESP reports funding from the Wellcome Trust (225925/Z/22/Z). EBB reports funding from the Wellcome Trust (224494/Z/21/Z). TC-S reports funding from the Royal Society (NIF∖R1∖231435). EFO reports a postdoctoral scholarship from the Conselho Nacional de Desenvolvimento Científico e Tecnológico (CNPq), Brazil and a research productivity fellowship from FUNDECT/CNPq. MLB reports a research fellowship from the Conselho Nacional de Desenvolvimento Científico e Tecnológico (CNPq), Brazil. All other authors declare no competing interests.
