# Supplementary material for: Zika exposure during pregnancy and the risk of hospitalisation and death due to community-acquired pneumonia in early childhood: a nationwide registry-based cohort study in Brazil
Source: Lancet Reg Health Am. 2026 Sep 30;64:101649. doi: 10.1016/j.lana.2026.101649 (PMC13654046; doi:10.1016/j.lana.2026.101649)
Supplement: Supplementary file 1 [file mmc1.pdf]

# Supplementary Appendix 1

**Zika exposure during pregnancy and the risk of hospitalization and death due to community-acquired pneumonia in early childhood: a nationwide registry-based cohort study in Brazil**

Everton Falcão de Oliveira, Thiago Cerqueira-Silva, João Guilherme G. Tedde, Bruno Holsback Uesato, José Victor Bortolotto Bampi, Elizabeth B. Brickley, Maria Gloria Teixeira, Mauricio L. Barreto, Enny S. Paixão

## Table of Contents

**Supplementary Material**. STOBRE Statement—Checklist of items that should be included in reports of cohort studies ........ 3

**Supplementary Figure 1**. Simplified Directed Acyclic Graph showing the hypothesized causal relationships between prenatal Zika virus exposure and morbimortality due to pneumonia ........ 6

**Supplementary Figure 2**. Covariate balance before and after Inverse Probability of Treatment Weighting (IPTW) based on prenatal Zika exposure (Zika-exposed CZS versus Zika-unexposed). Main Analysis: Multivariable Regression ........ 7

**Supplementary Figure 3**. Covariate balance before and after IPTW based on prenatal Zika exposure (Zika-exposed non-CZS versus Zika-unexposed). Main Analysis: Multivariable Regression ........ 8

**Supplementary Table 1**. Sample sizes before and after weighting (IPTW) based on prenatal Zika exposure (Zika-exposed CZS versus Zika-unexposed). *Main Analysis: Multivariable Regression* ........ 9

**Supplementary Table 2**. Covariate balance summary after IPTW based on prenatal Zika exposure. (Zika-exposed CZS versus Zika-unexposed). *Main Analysis: Multivariable Regression* ........ 9

**Supplementary Table 3**. Sample sizes before and after weighting (IPTW) based on prenatal Zika exposure (ZIKV-exposed non-CZS versus ZIKV-unexposed). *Main Analysis: Multivariable Regression* ........ 10

**Supplementary Table 4**. Covariate balance summary after IPTW based on prenatal Zika exposure (ZIKV-exposed non-CZS versus ZIKV-unexposed). *Main Analysis: Multivariable Regression* ........ 10

**Supplementary Table 5**. Baseline maternal and household characteristics of mothers included and not included in the *within sibling analysis* (ZIKV-exposed CZS versus ZIKV-unexposed) ........ 11

**Supplementary Table 6.** Baseline maternal and household characteristics of mothers included and not included in the *within sibling analysis* (ZIKV-exposed non-CZS versus ZIKV-unexposed) ........ 14

**Supplementary Figure 4**. Covariate balance before and after IPTW based on prenatal Zika exposure (Zika-exposed CZS versus Zika-unexposed). Sensitivity Analysis: exclusion of preterm births and low birth weight ........ 17

**Supplementary Figure 5**. Covariate balance before and after IPTW based on prenatal Zika exposure (Zika-exposed non-CZS versus Zika-unexposed). Sensitivity Analysis: exclusion of preterm births and low birth weight ........ 18

**Supplementary Figure 6**. Covariate balance before and after IPTW based on prenatal Zika exposure (Zika-exposed CZS versus Zika-unexposed). Sensitivity Analysis: Zika infection laboratory confirmed ........ 19

**Supplementary Figure 7**. Covariate balance before and after IPTW based on prenatal Zika exposure (Zika-exposed non-CZS versus Zika-unexposed). Sensitivity Analysis: Zika infection laboratory confirmed ........ 20

**Supplementary Table 7**. Sample sizes before and after weighting (IPTW) based on prenatal Zika exposure (Zika-exposed CZS versus Zika-unexposed). *Sensitivity Analysis: exclusion of preterm births and low birth weight* ........ 21

**Supplementary Table 8**. Covariate balance summary after IPTW based on prenatal Zika exposure (Zika-exposed CZS versus Zika-unexposed). *Sensitivity Analysis: exclusion of preterm births and low birth weight* ........ 21

**Supplementary Table 9**. Sample sizes before and after weighting (IPTW) based on prenatal Zika exposure (Zika-exposed non-CZS versus Zika-unexposed). *Sensitivity Analysis: exclusion of preterm births and low birth weight* ........ 22

**Supplementary Table 10**. Covariate balance summary after IPTW based on prenatal Zika exposure (Zika-exposed non-CZS versus Zika-unexposed). *Sensitivity Analysis: exclusion of preterm births and low birth weight* ........ 22

**Supplementary Table 11**. Sample sizes before and after weighting (IPTW) based on prenatal Zika exposure (Zika-exposed CZS versus Zika-unexposed). *Sensitivity Analysis: Zika infection laboratory confirmed* ........ 23

**Supplementary Table 12**. Covariate balance summary after IPTW based on prenatal Zika exposure (Zika-exposed CZS versus Zika-unexposed). *Sensitivity Analysis: Zika infection laboratory confirmed* ........ 23

**Supplementary Table 13**. Sample sizes before and after weighting (IPTW) based on prenatal Zika exposure (ZIKV-exposed non-CZS versus ZIKV-unexposed). *Sensitivity Analysis: Zika infection laboratory confirmed* ........ 23

**Supplementary Table 14**. Covariate balance summary after IPTW based on prenatal Zika exposure (Zika-exposed non-CZS versus Zika-unexposed). *Sensitivity Analysis: Zika infection laboratory confirmed* ........ 24

**Supplementary Table 15**. Sample sizes before and after matching (ZIKV-exposed CZS versus ZIKV-unexposed). *Sensitivity Analysis: exact and propensity score matching* ........ 24

**Supplementary Table 16**. Covariate balance summary after matching (ZIKV-exposed CZS versus ZIKV-unexposed). *Sensitivity Analysis: exact and propensity score matching* ........ 24

**Supplementary Table 17**. Sample sizes before and after matching (ZIKV-exposed non-CZS versus ZIKV-unexposed). *Sensitivity Analysis: exact and propensity score matching* ........ 25

**Supplementary Table 18**. Covariate balance summary (ZIKV-exposed non-CZS versus ZIKV-unexposed). *Sensitivity Analysis: exact and propensity score matching* . 25

**Supplementary Material**. STOBRE Statement—Checklist of items that should be included in reports of cohort studies

| | Item No | Recommendation | |
|---|---|---|---|
| **Title and abstract** | 1 | (*a*) Indicate the study's design with a commonly used term in the title or the abstract | Present in the title and in the abstract |
| | | (*b*) Provide in the abstract an informative and balanced summary of what was done and what was found | Present in the abstract (Pages 2 and 3) |
| **Introduction** | | | |
| Background/rationale | 2 | Explain the scientific background and rationale for the investigation being reported | Paragraph 3 (Introduction; pages 4 and 5) |
| Objectives | 3 | State specific objectives, including any prespecified hypotheses | Paragraph 3 (Introduction; pages 4 and 5) |
| **Methods** | | | |
| Study design | 4 | Present key elements of study design early in the paper | Section "*Study design and period*" (Methods; page 5) |
| Setting | 5 | Describe the setting, locations, and relevant dates, including periods of recruitment, exposure, follow-up, and data collection | Section "*Study design and period*" (Methods; page 5) |
| Participants | 6 | (*a*) Give the eligibility criteria, and the sources and methods of selection of participants. Describe methods of follow-up | Section "*Participants*" (Methods; pages 7 and 8) |
| | | (*b*) For matched studies, give matching criteria and number of exposed and unexposed | N/A |
| Variables | 7 | Clearly define all outcomes, exposures, predictors, potential confounders, and effect modifiers. Give diagnostic criteria, if applicable | Sections "*Exposure measures*", "*Outcome measures*", and "*Covariates*" (Methods; pages 8 to 10) |

| | | | |
|---|---|---|---|
| Data sources/ measurement | 8* | For each variable of interest, give sources of data and details of methods of assessment (measurement). Describe comparability of assessment methods if there is more than one group | Sections "*Data sources*", "*Exposure measures*", "*Outcome measures*", and "*Covariates*" (Methods; pages 6 and 7; 8 to 10) |
| Bias | 9 | Describe any efforts to address potential sources of bias | Section "*Statistical analysis*" (sensitivity analyses described in Methods, *Multivariable regression analyses*; pages 11 and 12) |
| Study size | 10 | Explain how the study size was arrived at | N/A |
| Quantitative variables | 11 | Explain how quantitative variables were handled in the analyses. If applicable, describe which groupings were chosen and why | Section "*Statistical analysis*" (Methods; pages 10 to 12) |
| Statistical methods | 12 | (*a*) Describe all statistical methods, including those used to control for confounding | Section "*Statistical analysis*" (Methods; pages 10 to 12) |
| | | (*b*) Describe any methods used to examine subgroups and interactions | |
| | | (*c*) Explain how missing data were addressed | Section "*Statistical analysis*" (Methods; page 11) |
| | | (*d*) If applicable, explain how loss to follow-up was addressed | Section "*Statistical analysis*" (Methods; page 10) |
| | | (*e*) Describe any sensitivity analyses | Section "*Statistical analysis*" (Methods; pages 11 to 12) |
| **Results** | | | |
| Participants | 13* | (a) Report numbers of individuals at each stage of study—eg numbers potentially eligible, examined for eligibility, confirmed eligible, included in the study, completing follow-up, and analysed | Paragraph 1 (Results, page 13) |
| | | (b) Give reasons for non-participation at each stage | Figure 1 (Results) |
| | | (c) Consider use of a flow diagram | Figure 1 (Results) |

| | | | |
|---|---|---|---|
| Descriptive data | 14* | (a) Give characteristics of study participants (eg demographic, clinical, social) and information on exposures and potential confounders | Paragraph 2 and Table 1 (Results, page 13) |
| | | (b) Indicate number of participants with missing data for each variable of interest | Table 1 (Results) |
| | | (c) Summarise follow-up time (eg, average and total amount) | Figure 2 (Results) |
| Outcome data | 15* | Report numbers of outcome events or summary measures over time | Paragraph 3, Table 1, and Figure 2 (Results, pages 13 and 14) |
| Main results | 16 | (*a*) Give unadjusted estimates and, if applicable, confounder-adjusted estimates and their precision (eg, 95% confidence interval). Make clear which confounders were adjusted for and why they were included | Paragraph 4 and Figure 3 (Results; page 14); Supplementary tables |
| | | (*b*) Report category boundaries when continuous variables were categorized | Paragraph 4 and Figure 3 (Results; page 14); Supplementary tables |
| | | (*c*) If relevant, consider translating estimates of relative risk into absolute risk for a meaningful time period | N/A |
| Other analyses | 17 | Report other analyses done—eg analyses of subgroups and interactions, and sensitivity analyses | Section "*Sensitivity analyses*" (Results; page 15) |
| **Discussion** | | | |
| Key results | 18 | Summarise key results with reference to study objectives | Paragraph 1 (Discussion; page 15) |
| Limitations | 19 | Discuss limitations of the study, taking into account sources of potential bias or imprecision. Discuss both direction and magnitude of any potential bias | Paragraphs 8 and 9 (Discussion; pages 18 and 19) |
| Interpretation | 20 | Give a cautious overall interpretation of results considering objectives, limitations, multiplicity of analyses, results from similar studies, and other relevant evidence | Paragraphs 2 to 6 (Discussion; pages 15 to 17) |
| Generalisability | 21 | Discuss the generalisability (external validity) of the study results | Paragraph 9 (Discussion; page 19) |
| **Other information** | | | |
| Funding | 22 | Give the source of funding and the role of the funders for the present study and, if applicable, for the original study on which the present article is based | Role of funding source (page 13); Acknowledgements (page 20) |

**Supplementary Figure 1**. Simplified Directed Acyclic Graph showing the hypothesized causal relationships between prenatal Zika virus exposure and morbimortality due to pneumonia

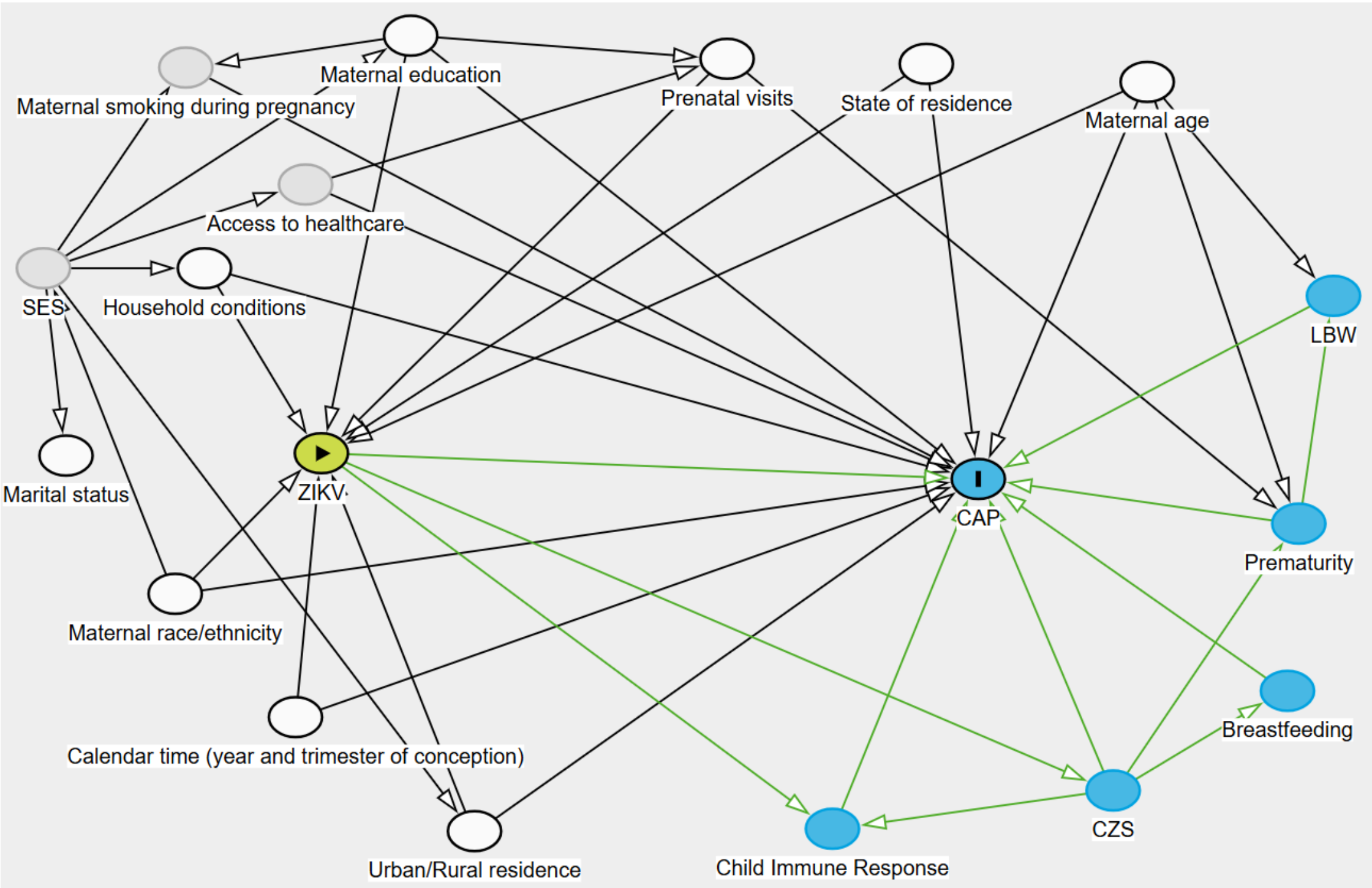


White: adjusted covariates; Blue: mediator (not included in the adjusted models); Grey: unmeasured covariate.
ZIKV: Zika virus (prenatal exposure to Zika virus); CAP: Community-acquired pneumonia (during childhood); SES: Socioeconomic status; LBW: Low birth weight; CZS: Congenital Zika Syndrome.

**Supplementary Figure 2**. Covariate balance before and after Inverse Probability of Treatment Weighting (IPTW) based on prenatal Zika exposure (Zika-exposed CZS versus Zika-unexposed). Main Analysis: Multivariable Regression

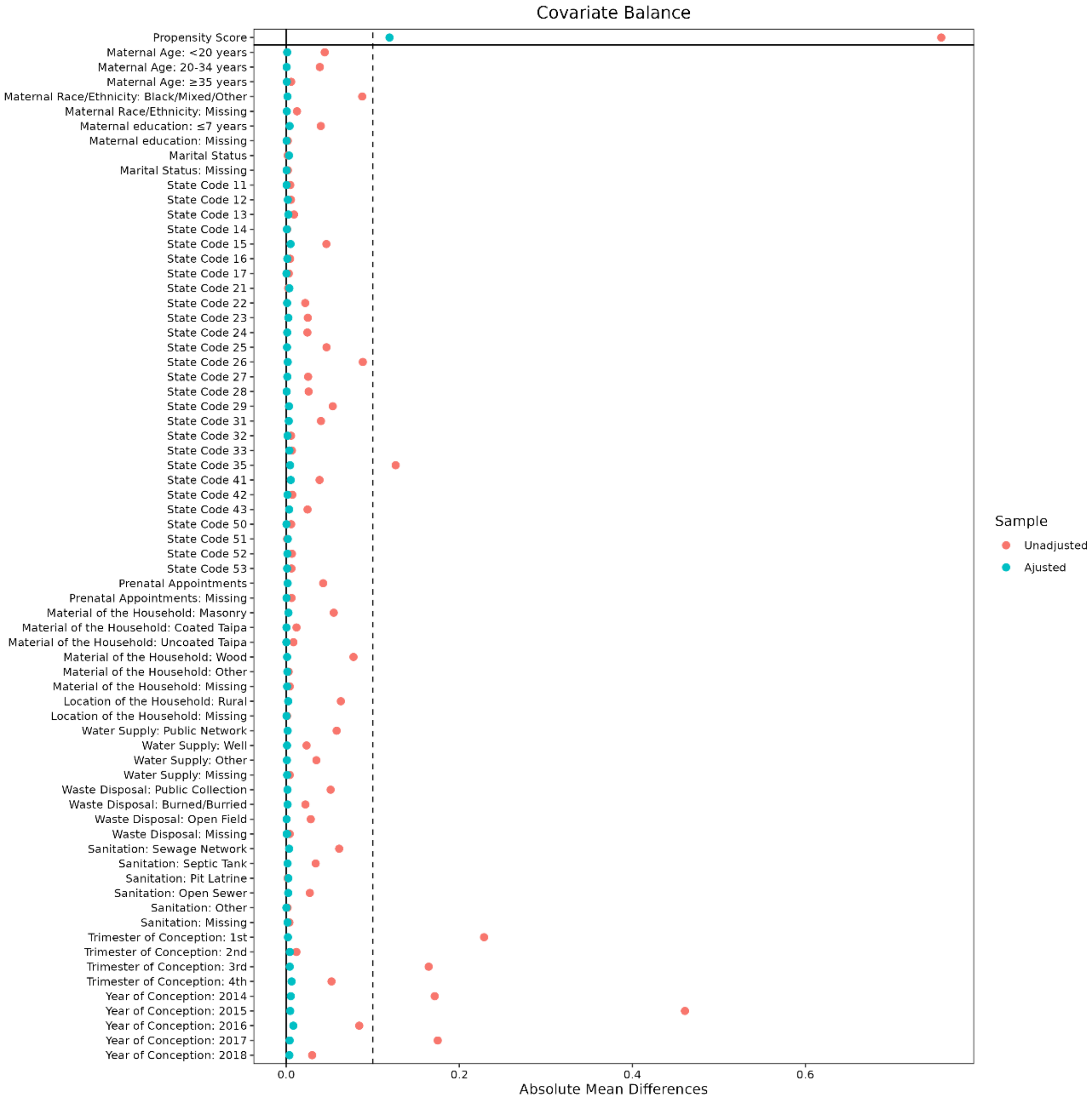

**Supplementary Figure 3**. Covariate balance before and after IPTW based on prenatal Zika exposure (Zika-exposed non-CZS versus Zika-unexposed). Main Analysis: Multivariable Regression

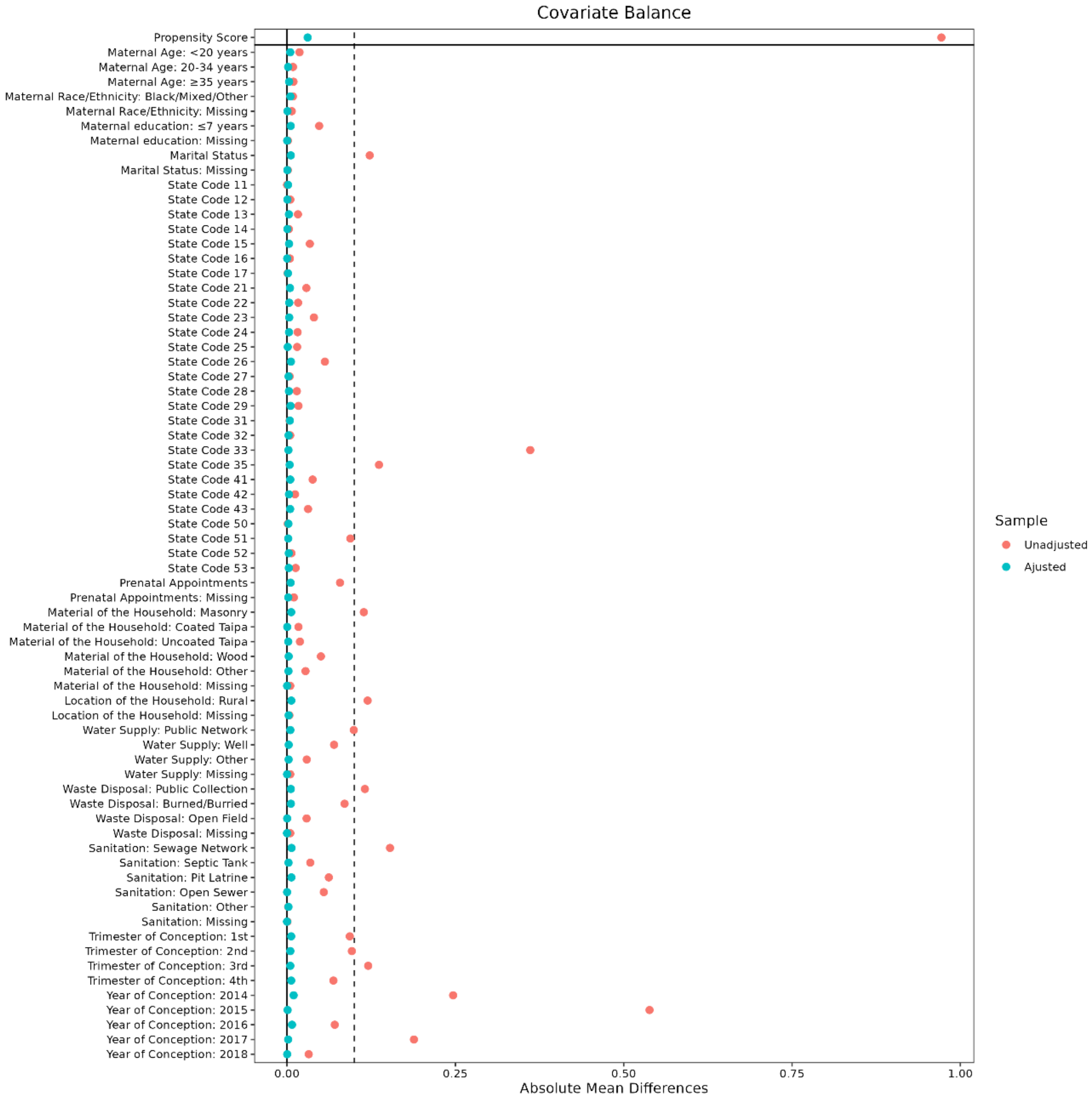

**Supplementary Table 1**. Sample sizes before and after weighting (IPTW) based on prenatal Zika exposure (Zika-exposed CZS versus Zika-unexposed). *Main Analysis: Multivariable Regression*

| **Sample** | **Control** | **Treated** |
|---|---|---|
| Original sample | 4,108,409 | 1,786 |
| Weighted (Unadjusted N)* | 4,108,409 | 1,786 |
| Weighted (Effective Sample Size) | 735,597.6 | 1,786 |

*Unadjusted N corresponds to the observed sample size prior to weighting.

**Supplementary Table 2**. Covariate balance summary after IPTW based on prenatal Zika exposure. (Zika-exposed CZS versus Zika-unexposed). *Main Analysis: Multivariable Regression*

| **Metric** | **Value** |
|---|---|
| Number of covariates | 70 |
| Balanced (absolute SMD < 0.1) | 70 |
| Not balanced (absolute SMD ≥ 0.1) | 0 |
| Largest adjusted SMD (Conception year: 2016) | 0.0081 |

SMD: Standardized Mean Difference

**Supplementary Table 3**. Sample sizes before and after weighting (IPTW) based on prenatal Zika exposure (ZIKV-exposed non-CZS versus ZIKV-unexposed). *Main Analysis: Multivariable Regression*

| Sample | Control | Treated |
|---|---|---|
| Original sample | 4,108,409 | 3,900 |
| Weighted (Unadjusted N)* | 4,108,409 | 3,900 |
| Weighted (Effective Sample Size) | 259,871.1 | 3,900 |

*Unadjusted N corresponds to the observed sample size prior to weighting.

**Supplementary Table 4**. Covariate balance summary after IPTW based on prenatal Zika exposure (ZIKV-exposed non-CZS versus ZIKV-unexposed). *Main Analysis: Multivariable Regression*

| Metric | Value |
|---|---|
| Number of covariates | 71 |
| Balanced (absolute SMD < 0.1) | 71 |
| Not balanced (absolute SMD ≥ 0.1) | 0 |
| Largest adjusted SMD (Conception year: 2014) | 0.0098 |

SMD: Standardized Mean Difference

**Supplementary Table 5**. Baseline maternal and household characteristics of mothers included and not included in the *within sibling analysis* (ZIKV-exposed CZS versus ZIKV-unexposed)

| **Characteristic** | **CZS mother not included** N = 1,568 | **Included in sibling analysis** N = 218 |
|---|---|---|
| **Age (years)**, mean (SD) | 25.0 (6.7) | 22.9 (5.7) |
| **Age** | | |
| <20 years | 384.0 (24.5%) | 78.0 (35.8%) |
| 20-34 years | 1,025.0 (65.4%) | 130.0 (59.6%) |
| ≥35 years | 159.0 (10.1%) | 10.0 (4.6%) |
| **Educational attainment** | | |
| ≤7 years | 423.0 (27.0%) | 77.0 (35.3%) |
| >7 years | 1,122.0 (71.6%) | 137.0 (62.8%) |
| Missing | 23.0 (1.5%) | 4.0 (1.8%) |
| **Race/Ethnicity** | | |
| Black/Mixed/Other | 1,243.0 (79.3%) | 176.0 (80.7%) |
| White | 234.0 (14.9%) | 30.0 (13.8%) |
| Missing | 91.0 (5.8%) | 12.0 (5.5%) |
| **Marital status** | | |
| In a relationship | 730.0 (46.6%) | 89.0 (40.8%) |
| Single | 819.0 (52.2%) | 128.0 (58.7%) |
| Missing | 19.0 (1.2%) | 1.0 (0.5%) |
| **Prenatal appointments** | | |
| Inadequate | 392.0 (25.0%) | 67.0 (30.7%) |
| Adequate | 1,130.0 (72.1%) | 145.0 (66.5%) |
| Missing | 46.0 (2.9%) | 6.0 (2.8%) |
| **Location** | | |
| Urban | 1,126.0 (71.8%) | 170.0 (78.0%) |
| Rural | 392.0 (25.0%) | 44.0 (20.2%) |

| Characteristic | CZS mother not included<br>N = 1,568 | Included in sibling analysis<br>N = 218 |
|---|---|---|
| Missing | 50.0 (3.2%) | 4.0 (1.8%) |
| **Geographic region of Brazil** | | |
| Southeast | 328.0 (20.9%) | 41.0 (18.8%) |
| South | 21.0 (1.3%) | 3.0 (1.4%) |
| Central West | 106.0 (6.8%) | 14.0 (6.4%) |
| Northeast | 1,043.0 (66.5%) | 143.0 (65.6%) |
| North | 70.0 (4.5%) | 17.0 (7.8%) |
| **Household water supply** | | |
| Public network | 1,016.0 (64.8%) | 141.0 (64.7%) |
| Well/Spring | 318.0 (20.3%) | 39.0 (17.9%) |
| Other | 166.0 (10.6%) | 31.0 (14.2%) |
| Missing | 68.0 (4.3%) | 7.0 (3.2%) |
| **Household waste disposal** | | |
| Collected | 1,103.0 (70.3%) | 161.0 (73.9%) |
| Burned/ Buried | 250.0 (15.9%) | 33.0 (15.1%) |
| Open-air dump | 127.0 (8.1%) | 16.0 (7.3%) |
| Other | 20.0 (1.3%) | 1.0 (0.5%) |
| Missing | 68.0 (4.3%) | 7.0 (3.2%) |
| **Household sewage system** | | |
| Public sewer network | 633.0 (40.4%) | 92.0 (42.2%) |
| Septic tank | 261.0 (16.6%) | 34.0 (15.6%) |
| Rudimentary cesspit | 338.0 (21.6%) | 51.0 (23.4%) |
| Ditch | 224.0 (14.3%) | 25.0 (11.5%) |
| Other | 27.0 (1.7%) | 5.0 (2.3%) |
| Missing | 85.0 (5.4%) | 11.0 (5.0%) |
| **Housing construction material** | | |
| Masonry | 1,248.0 (79.6%) | 168.0 (77.1%) |

| **Characteristic** | **CZS mother not included**<br>N = 1,568 | **Included in sibling analysis**<br>N = 218 |
|---|---|---|
| Covered wattle and daub (*taipa*) | 59.0 (3.8%) | 7.0 (3.2%) |
| Uncovered wattle and daub (*taipa*) | 55.0 (3.5%) | 5.0 (2.3%) |
| Wood | 52.0 (3.3%) | 15.0 (6.9%) |
| Other | 86.0 (5.5%) | 16.0 (7.3%) |
| Missing | 68.0 (4.3%) | 7.0 (3.2%) |

Note. Characteristics were defined based on the first pregnancy recorded during the study period (2015–2018).

**Supplementary Table 6.** Baseline maternal and household characteristics of mothers included and not included in the *within sibling analysis* (ZIKV-exposed non-CZS versus ZIKV-unexposed)

| Characteristic | **ZIKV-exposed mother not included**<br>N = 3,643 | **Included in Sibling Analysis**<br>N = 256 |
|---|---|---|
| **Age (years)**, mean (SD) | 25.8 (6.5) | 23.8 (5.9) |
| **Age** | | |
| <20 years | 678.0 (18.6%) | 76.0 (29.7%) |
| 20-34 years | 2,549.0 (70.0%) | 166.0 (64.8%) |
| ≥35 years | 416.0 (11.4%) | 14.0 (5.5%) |
| **Educational attainment** | | |
| ≤7 years | 2,903.0 (79.7%) | 185.0 (72.3%) |
| >7 years | 696.0 (19.1%) | 70.0 (27.3%) |
| Missing | 44.0 (1.2%) | 1.0 (0.4%) |
| **Race/Ethnicity** | | |
| Black/Mixed/Other | 2,668.0 (73.2%) | 198.0 (77.3%) |
| White | 831.0 (22.8%) | 52.0 (20.3%) |
| Missing | 144.0 (4.0%) | 6.0 (2.3%) |
| **Marital status** | | |
| In a relationship | 1,225.0 (33.6%) | 87.0 (34.0%) |
| Single | 2,385.0 (65.5%) | 169.0 (66.0%) |
| Missing | 33.0 (0.9%) | 0.0 (0.0%) |
| **Prenatal appointments** | | |
| Inadequate | 503.0 (13.8%) | 60.0 (23.4%) |
| Adequate | 3,096.0 (85.0%) | 189.0 (73.8%) |
| Missing | 44.0 (1.2%) | 7.0 (2.7%) |
| **Location** | | |
| Urban | 3,283.0 (90.1%) | 228.0 (89.1%) |
| Rural | 238.0 (6.5%) | 22.0 (8.6%) |

| Characteristic | ZIKV-exposed mother not included<br>N = 3,643 | Included in Sibling Analysis<br>N = 256 |
|---|---|---|
| Missing | 122.0 (3.3%) | 6.0 (2.3%) |
| **Geographic region of Brazil** | | |
| Southeast | 2,156.0 (59.2%) | 124.0 (48.4%) |
| South | 8.0 (0.2%) | 0.0 (0.0%) |
| Central West | 617.0 (16.9%) | 50.0 (19.5%) |
| Northeast | 534.0 (14.7%) | 53.0 (20.7%) |
| North | 328.0 (9.0%) | 29.0 (11.3%) |
| **Household water supply** | | |
| Public network | 2,889.0 (79.3%) | 196.0 (76.6%) |
| Well/Spring | 396.0 (10.9%) | 31.0 (12.1%) |
| Other | 170.0 (4.7%) | 19.0 (7.4%) |
| Missing | 188.0 (5.2%) | 10.0 (3.9%) |
| **Household waste disposal** | | |
| Collected | 3,136.0 (86.1%) | 216.0 (84.4%) |
| Burned/ Buried | 192.0 (5.3%) | 22.0 (8.6%) |
| Open-air dump | 91.0 (2.5%) | 6.0 (2.3%) |
| Other | 36.0 (1.0%) | 2.0 (0.8%) |
| Missing | 188.0 (5.2%) | 10.0 (3.9%) |
| **Household sewage system** | | |
| Public sewer network | 2,217.0 (60.9%) | 148.0 (57.8%) |
| Septic tank | 372.0 (10.2%) | 18.0 (7.0%) |
| Rudimentary cesspit | 575.0 (15.8%) | 49.0 (19.1%) |
| Ditch | 217.0 (6.0%) | 24.0 (9.4%) |
| Other | 54.0 (1.5%) | 4.0 (1.6%) |
| Missing | 208.0 (5.7%) | 13.0 (5.1%) |
| **Housing construction material** | | |
| Masonry | 3,078.0 (84.5%) | 204.0 (79.7%) |

| **Characteristic** | **ZIKV-exposed mother not included**<br>N = 3,643 | **Included in Sibling Analysis**<br>N = 256 |
| --- | --- | --- |
| Covered wattle and daub (*taipa*) | 33.0 (0.9%) | 3.0 (1.2%) |
| Uncovered wattle and daub (*taipa*) | 24.0 (0.7%) | 4.0 (1.6%) |
| Wood | 216.0 (5.9%) | 30.0 (11.7%) |
| Other | 104.0 (2.9%) | 5.0 (2.0%) |
| Missing | 188.0 (5.2%) | 10.0 (3.9%) |

Note. Characteristics were defined based on the first pregnancy recorded during the study period (2015–2018).

**Supplementary Figure 4**. Covariate balance before and after IPTW based on prenatal Zika exposure (Zika-exposed CZS versus Zika-unexposed). Sensitivity Analysis: exclusion of preterm births and low birth weight

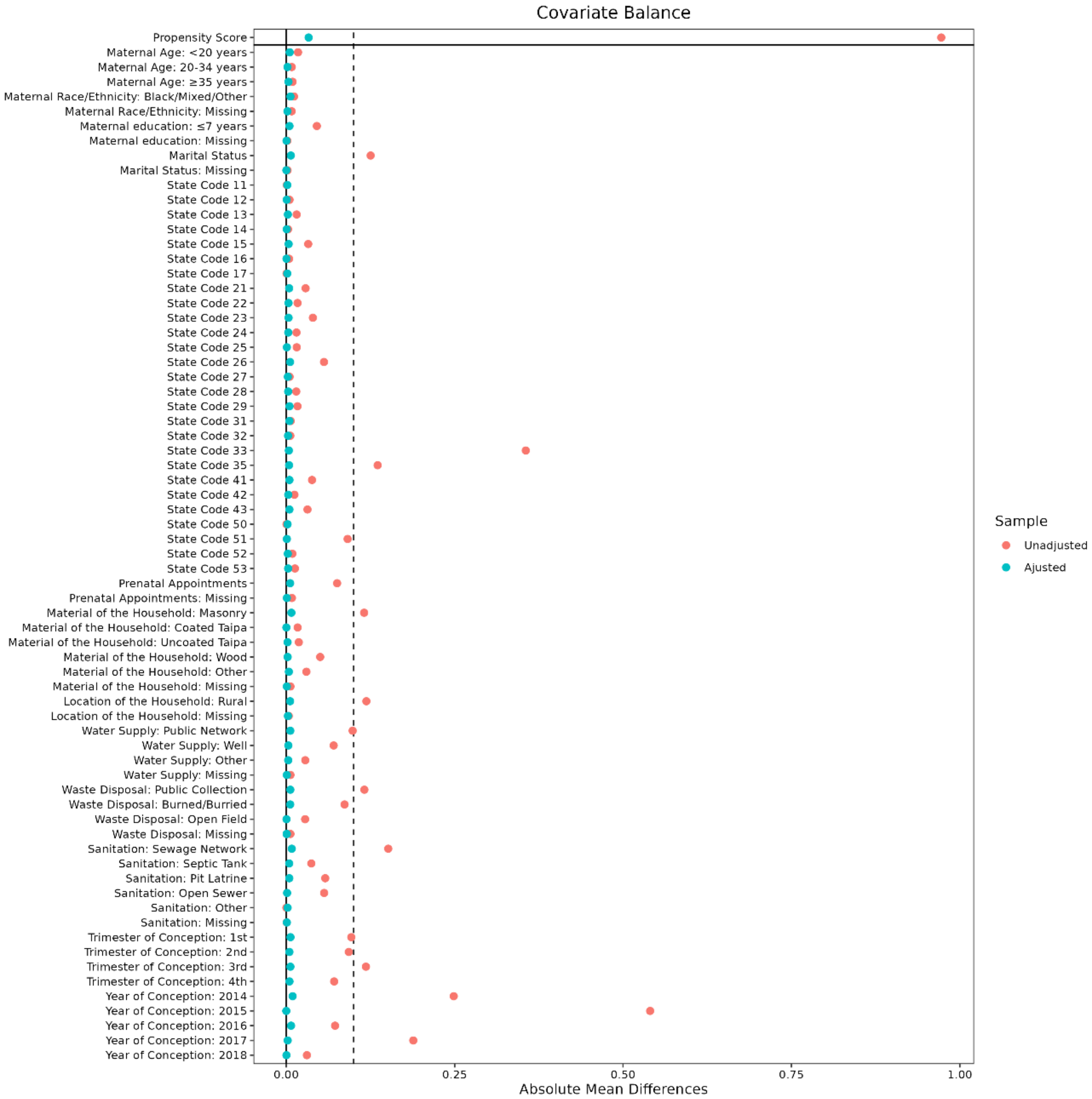

**Supplementary Figure 5**. Covariate balance before and after IPTW based on prenatal Zika exposure (Zika-exposed non-CZS versus Zika-unexposed). Sensitivity Analysis: exclusion of preterm births and low birth weight

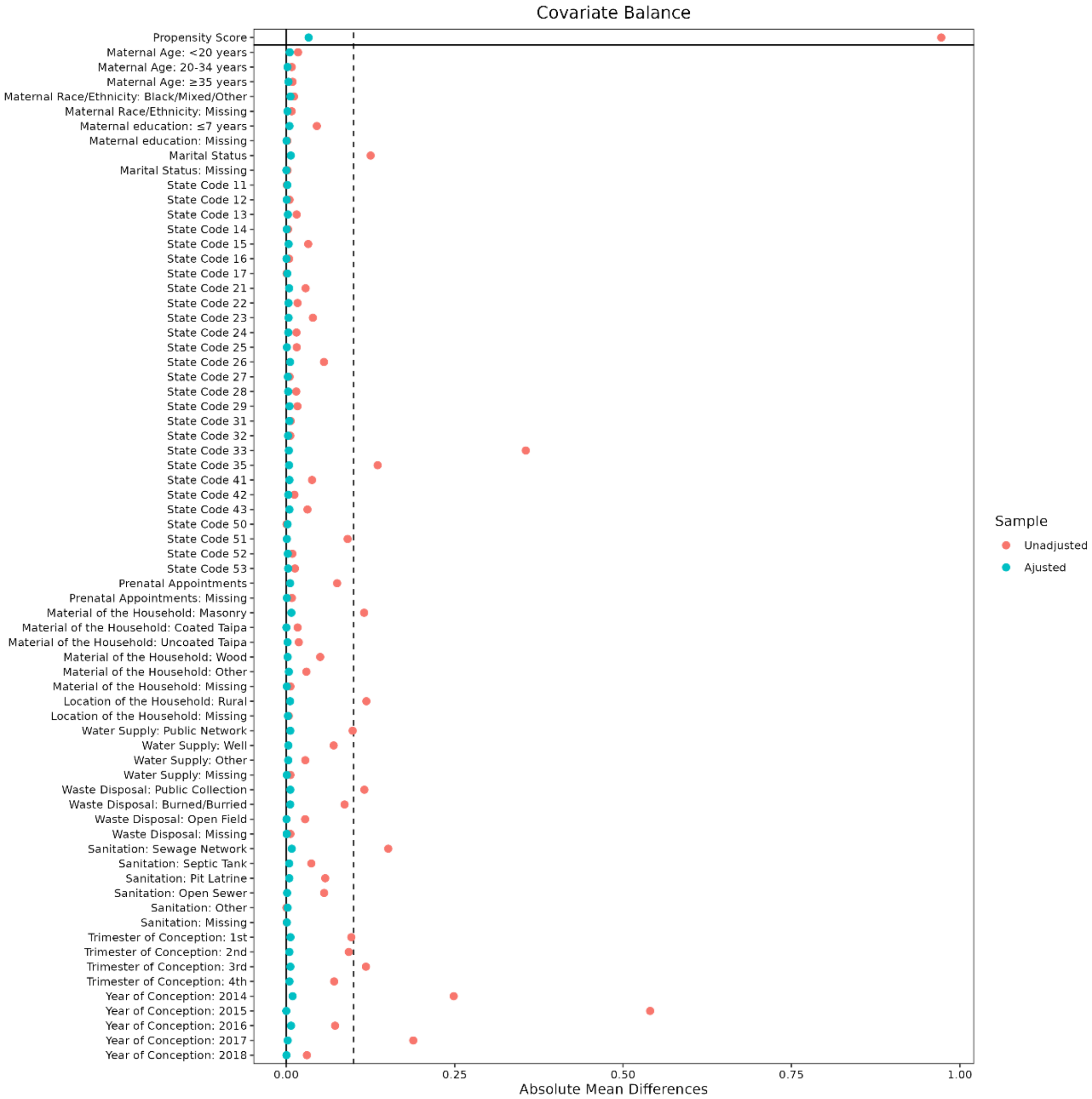

**Supplementary Figure 6**. Covariate balance before and after IPTW based on prenatal Zika exposure (Zika-exposed CZS versus Zika-unexposed). Sensitivity Analysis: Zika infection laboratory confirmed

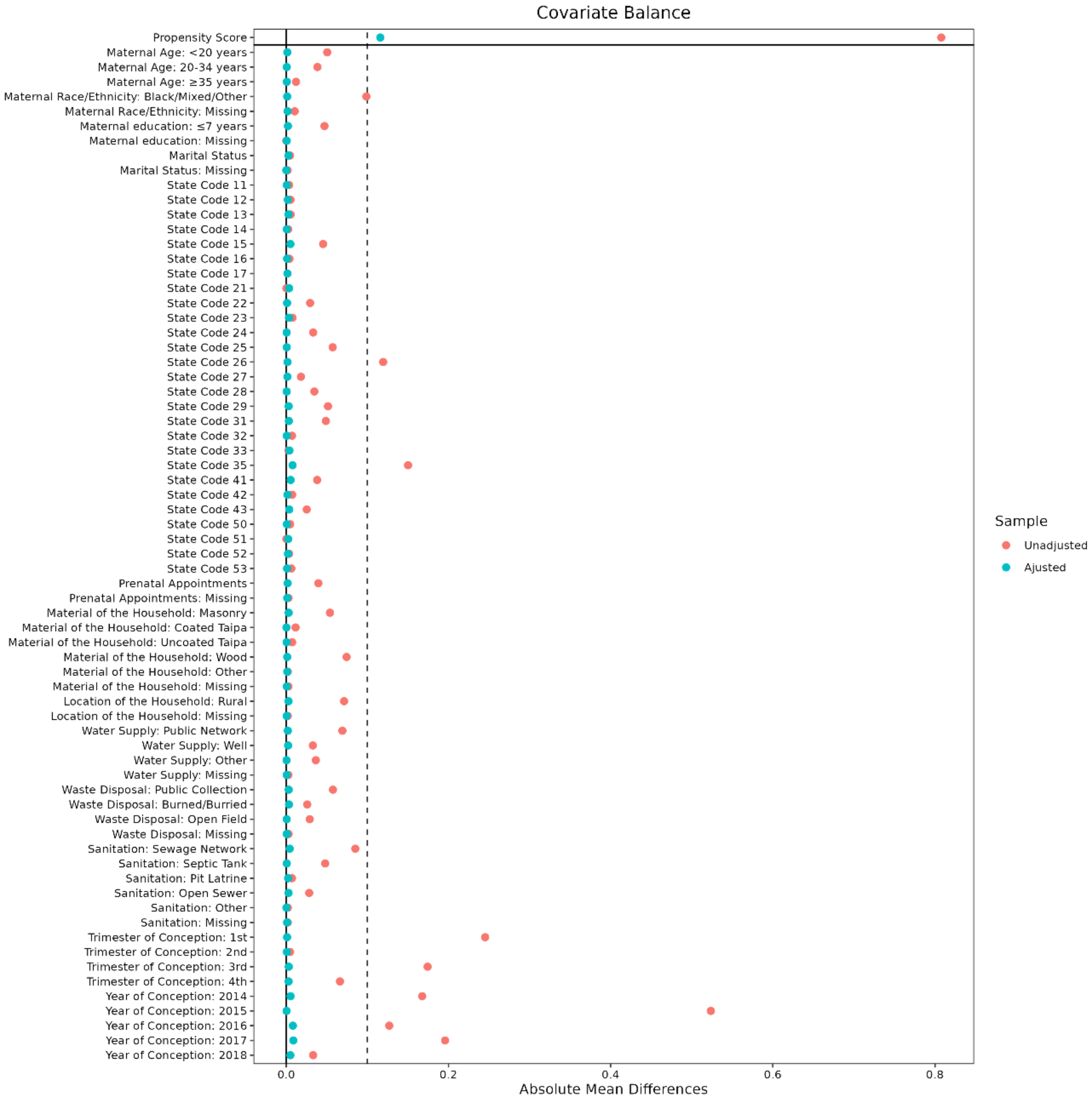

**Supplementary Figure 7**. Covariate balance before and after IPTW based on prenatal Zika exposure (Zika-exposed non-CZS versus Zika-unexposed). Sensitivity Analysis: Zika infection laboratory confirmed

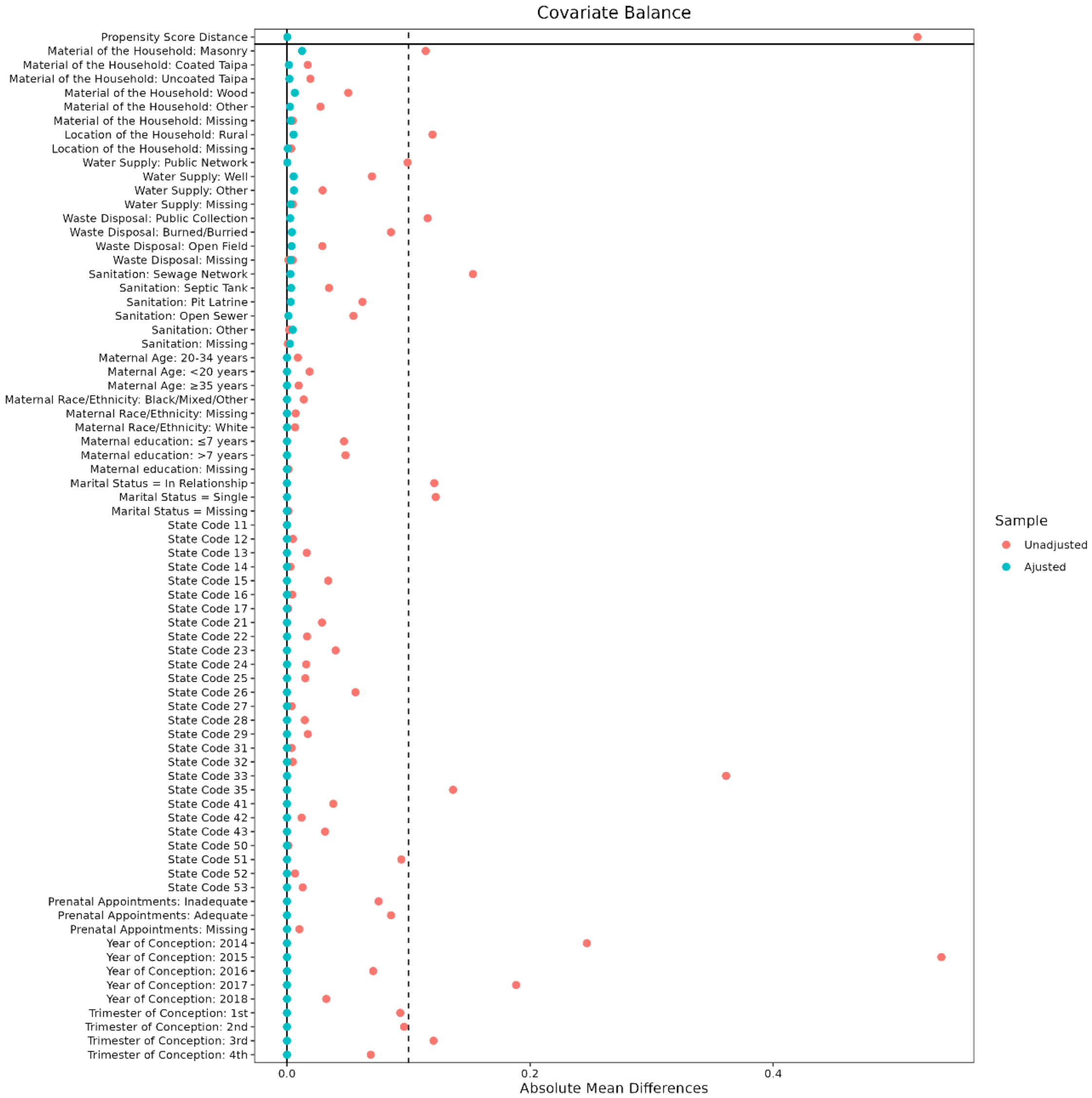

**Supplementary Table 7**. Sample sizes before and after weighting (IPTW) based on prenatal Zika exposure (Zika-exposed CZS versus Zika-unexposed). *Sensitivity Analysis: exclusion of preterm births and low birth weight*

| Sample | Control | Treated |
|---|---|---|
| Original sample | 3,548,637 | 1,062 |
| Weighted (Unadjusted N)* | 3,548,637 | 1,062 |
| Weighted (Effective Sample Size) | 551,367.7 | 1,062 |

*Unadjusted N corresponds to the observed sample size prior to weighting.

**Supplementary Table 8**. Covariate balance summary after IPTW based on prenatal Zika exposure (Zika-exposed CZS versus Zika-unexposed). *Sensitivity Analysis: exclusion of preterm births and low birth weight*

| Metric | Value |
|---|---|
| Number of covariates | 70 |
| Balanced (absolute SMD < 0.1) | 70 |
| Not balanced (absolute SMD ≥ 0.1) | 0 |
| Largest adjusted SMD (Conception year: 2016) | 0.0097 |

SMD: Standardized Mean Difference

**Supplementary Table 9**. Sample sizes before and after weighting (IPTW) based on prenatal Zika exposure (Zika-exposed non-CZS versus Zika-unexposed). *Sensitivity Analysis: exclusion of preterm births and low birth weight*

| Sample | Control | Treated |
|---|---|---|
| Original sample | 3,548,637 | 3,388 |
| Weighted (Unadjusted N)* | 3,548,637 | 3,388 |
| Weighted (Effective Sample Size) | 231,202.5 | 3,388 |

*Unadjusted N corresponds to the observed sample size prior to weighting.

**Supplementary Table 10**. Covariate balance summary after IPTW based on prenatal Zika exposure (Zika-exposed non-CZS versus Zika-unexposed). *Sensitivity Analysis: exclusion of preterm births and low birth weight*

| Metric | Value |
|---|---|
| Number of covariates | 71 |
| Balanced (absolute SMD < 0.1) | 71 |
| Not balanced (absolute SMD ≥ 0.1) | 0 |
| Largest adjusted SMD (Conception year: 2014) | 0.0094 |

SMD: Standardized Mean Difference

**Supplementary Table 11**. Sample sizes before and after weighting (IPTW) based on prenatal Zika exposure (Zika-exposed CZS versus Zika-unexposed). *Sensitivity Analysis: Zika infection laboratory confirmed*

| Sample | Control | Treated |
|---|---|---|
| Original sample | 4,108,615 | 1,477 |
| Weighted (Unadjusted N)* | 4,108,615 | 1,477 |
| Weighted (Effective Sample Size) | 527,287.1 | 1,477 |

*Unadjusted N corresponds to the observed sample size prior to weighting.

**Supplementary Table 12**. Covariate balance summary after IPTW based on prenatal Zika exposure (Zika-exposed CZS versus Zika-unexposed). *Sensitivity Analysis: Zika infection laboratory confirmed*

| Metric | Value |
|---|---|
| Number of covariates | 70 |
| Balanced (absolute SMD < 0.1) | 70 |
| Not balanced (absolute SMD ≥ 0.1) | 0 |
| Largest adjusted SMD (Conception year: 2017) | 0.0087 |

SMD: Standardized Mean Difference

**Supplementary Table 13**. Sample sizes before and after weighting (IPTW) based on prenatal Zika exposure (ZIKV-exposed non-CZS versus ZIKV-unexposed). *Sensitivity Analysis: Zika infection laboratory confirmed*

| Sample | Control | Treated |
|---|---|---|
| Original sample | 4,108,615 | 1,643 |
| Weighted (Unadjusted N)* | 4,108,615 | 1,643 |
| Weighted (Effective Sample Size) | 281,410.8 | 1,643 |

*Unadjusted N corresponds to the observed sample size prior to weighting.

**Supplementary Table 14**. Covariate balance summary after IPTW based on prenatal Zika exposure (Zika-exposed non-CZS versus Zika-unexposed). *Sensitivity Analysis: Zika infection laboratory confirmed*

| Metric | Value |
|---|---|
| Number of covariates | 71 |
| Balanced (absolute SMD < 0.1) | 71 |
| Not balanced (absolute SMD ≥ 0.1) | 0 |
| Largest adjusted SMD (Conception year: 2014) | 0.0143 |

SMD: Standardized Mean Difference

**Supplementary Table 15**. Sample sizes before and after matching (ZIKV-exposed CZS versus ZIKV-unexposed). *Sensitivity Analysis: exact and propensity score matching*

| Sample | Control | Treated |
|---|---|---|
| Original sample | 4,108,409 | 1,786 |
| Matched (Unweighted) | 6,709 | 1,734 |
| Matched (Effective Sample Size) | 6,315.6 | 1,734 |
| Unmatched | 4,101,700 | 52 |

**Supplementary Table 16**. Covariate balance summary after matching (ZIKV-exposed CZS versus ZIKV-unexposed). *Sensitivity Analysis: exact and propensity score matching*

| Metric | Value |
|---|---|
| Number of covariates | 75 |
| Balanced (absolute SMD < 0.1) | 75 |
| Not balanced (absolute SMD ≥ 0.1) | 0 |
| Largest adjusted SMD (Material of the Household: masonry) | 0.019 |

SMD: Standardized Mean Difference

**Supplementary Table 17**. Sample sizes before and after matching (ZIKV-exposed non-CZS versus ZIKV-unexposed). *Sensitivity Analysis: exact and propensity score matching*

| **Sample** | **Control** | **Treated** |
|---|---|---|
| Original sample | 4,108,409 | 3,900 |
| Matched (Unweighted) | 15,190 | 3,850 |
| Matched (Effective Sample Size) | 14,783.1 | 3,850 |
| Unmatched | 4,093,219 | 50 |

**Supplementary Table 18**. Covariate balance summary (ZIKV-exposed non-CZS versus ZIKV-unexposed). *Sensitivity Analysis: exact and propensity score matching*

| **Metric** | **Value** |
|---|---|
| Number of covariates | 75 |
| Balanced (SMD < 0.1) | 75 |
| Not balanced (SMD ≥ 0.1) | 0 |
| Largest adjusted SMD (Material of the Household: masonry) | 0.012 |

SMD: Standardized Mean Difference
